# An open label study of the safety and efficacy of a single dose of weekly chloroquine and azithromycin administered for malaria prophylaxis in healthy adults challenged with 7G8 chloroquine-resistant *Plasmodium falciparum* in a controlled human malaria infection model

**DOI:** 10.1186/s12936-020-03409-z

**Published:** 2020-09-16

**Authors:** Jeffrey Livezey, Patrick Twomey, Meshell Morrison, Susan Cicatelli, Elizabeth H. Duncan, Melinda Hamer, Christine Lee, Jack Hutter, Kristin Mills, Jesse DeLuca, Lucas Poon, Daniel Selig, Chau Vuong, Jason Sousa, Thomas Oliver, Jason Bennett, James E. Moon, April Sikaffy, Martha Sedegah, Donna Tosh, Mara Kreishman-Deitrick, Paige Waterman

**Affiliations:** 1 Uniformed Services University of the Health Sciences, 4301 Jones Bridge Rd, Bethesda, MD 20814 USA; 2 Walter Reed Army Institute of Research, 503 Robert Grant Ave, Silver Spring, MD 20910 USA; 3 Naval Medical Research Center, 503 Robert Grant Ave, Silver Spring, MD 20910 USA

**Keywords:** Malaria chemoprophylaxis, Azithromycin, Chloroquine, Controlled human malaria infection

## Abstract

**Background:**

Malaria remains the top infectious disease threat facing the U.S. military in many forward operating environments. Compliance with malaria chemoprophylaxis remains a critical component in preventing malaria in the deployed Service Member. Studies of previous military operations show that compliance is consistently higher with weekly *versus* daily dosing regimens. Current FDA approved weekly chemoprophylaxis options have contraindications that can limit prescribing. The combination of chloroquine (CQ) with azithromycin (AZ) has previously been shown to be an efficacious treatment option for malaria, has pharmacokinetics compatible with weekly dosing, and has shown synergy when combined in vitro.

**Methods:**

In this open label study, 18 healthy volunteers, aged 18–50 years (inclusive), were randomly assigned to receive either 300 mg CQ or 300 mg CQ and 2 gm azithromycin (CQAZ) of directly observed therapy, weekly for 3 weeks prior to undergoing mosquito bite challenge with chloroquine-resistant *Plasmodium falciparum*. Volunteers that remained asymptomatic and had no evidence of parasitaemia continued to receive weekly post-exposure chemoprophylaxis for 3 weeks following malaria challenge. The primary endpoint was the number of volunteers that remained asymptomatic and had no evidence of parasitaemia 28 days after the malaria challenge.

**Results:**

All 6 (100%) volunteers randomized to the CQ control group became symptomatic with parasitaemia during the 28-day post-challenge period. Only 1/12 (8.3%) of volunteers in the CQAZ group developed symptoms and parasitaemia during the 28-day post-challenge period. However, after chemoprophylaxis was discontinued an additional 6 volunteers developed parasitaemia between days 28–41 after challenge, with 4 of 6 experiencing symptoms. 80% of subjects in the CQAZ group experienced treatment related gastrointestinal adverse events (including 13% that experienced severe nausea) compared to 38% in the CQ group. A comparison of the pharmacokinetics in the CQAZ group demonstrated higher azithromycin Cmax (p = 0.03) and AUC (p = 0.044) levels in those volunteers who never became parasitaemic compared to those who did.

**Conclusion:**

Given the high rate of side effects and poor efficacy when administered for 3 weeks before and after challenge, the combination of weekly chloroquine and azithromycin is a suboptimal regimen combination for weekly malaria chemoprophylaxis.

*Trial registration* ClinicalTrials.gov NCT03278808

## Background

Although the incidence rate has been declining since 2010, in 2018, there were still an estimated 228 million cases of malaria occurred worldwide [95% confidence interval (CI): 206–258 million] [1]. Further, there is a trend of increasing numbers of malaria cases in the United States (US), with over 2000 cases in 2016. Most of these cases were attributed to travel in endemic countries [2]. The most effective method to obviate malaria mortality and morbidity in travellers is an effective chemoprophylaxis regimen. There are currently 5 drugs licensed for a therapeutic indication of malaria prophylaxis by the US Food and Drug Administration (FDA): chloroquine, doxycycline, mefloquine, atovaquone–proguanil and the recently approved tafenoquine. Each of these medications has dosing frequency or side effect profile liabilities that limit the widespread usage, compliance and/or effectiveness required by organizations, such as the US military during deployments. CQ usage as a standalone chemoprophylactic has diminished greatly in many endemic areas due to resistance. Doxycycline’s high rates of gastrointestinal side effects, photosensitivity reactions and its daily dosing requirement, provide significant impairments to compliance. Saunders et al. showed only a 60% compliance rate for over 2000 deployed U.S. military personnel surveyed [3]. Although weekly dosed in 2002, the FDA and Roche Pharmaceuticals strengthened warnings concerning neuropsychiatric adverse reactions in the mefloquine drug label. Atovaquone–proguanil, while more tolerable than doxycycline, is more expensive, and has a daily dosing requirement.

Tafenoquine has the benefits of weekly dosing along with activity against all malaria stages that make it a promising prophylactic agent. However it is still new to the market and is contraindicated in those with unknown or deficient glucose-6-phosphate dehydrogenase activity [4]. With regards to use during pregnancy and fetal risk, these drugs run the spectrum from safe to use (CQ, mefloquine), currently unknown (atovaquone–proguanil, tafenoquine) to contraindicated (doxycycline). The combination of AZ and CQ presents the possibility of a low cost, weekly agent with a known, tolerable side effect profile with considerable safety data already available for both drugs’ use in pregnancy.

Azithromycin’s anti-malarial activity has been known for several years and mechanistically is believed to work by inhibiting apicoplast function [5]. Given the slow mechanism of action, AZ has also been investigated as a potential combination with a number of other faster acting anti-malarials [6]. In vitro, AZ showed additive to synergistic activity against eight CQ resistant *P falciparum* isolates [7]. In addition, susceptibility testing of CQAZ combinations against *P. falciparum* field isolates from Mali showed some synergy between CQ and AZ at the IC90 level and an additive effect at the IC50 level [8]. Furthermore, human trials showed no significant pharmacokinetic interaction when AZ was co-administered with CQ [9].

Azithromycin has been tried as a sole agent for malaria prophylaxis in clinical trials with modest effectiveness. Studies done in Kenya and Indonesia demonstrated modest results ranging from 72 to 84% efficacy with daily administration and 64% with weekly [10–12]. The combination of CQ and AZ has also been investigated as potential treatment for uncomplicated *P. falciparum*.

In India, 63 subjects received a combination of AZ (1gm) and CQ (1500 mg) for 3 days and saw a 97% resolution of fever and parasitaemia by day 7 with no relapse by Day 28. Although performed in areas of high *P. falciparum* CQ resistance, no testing was reported [13]. Two studies from multiple countries in Africa examined 1 gm AZ and 600 mg CQ base daily for 3 days for uncomplicated *P. falciparum*. Over 98% of subjects met the primary endpoint of being PCR confirmed parasite free at Day 28. In addition, in vitro analysis of study isolates of two countries showed rates of CQ resistance of 21% (Zambia) and 96% (Uganda), respectively. Serious adverse event (SAE) rates for CQAZ were four-fold lower than in the comparison treatment arm, mefloquine [14]. A subsequent *P. falciparum*, multi-country (India, Suriname, Colombia) treatment study in 2017 examined multiple AZ dosages (500 mg, 1 gm, 2 gm) combined with 600 mg base CQ for 3 days. The 500 mg AZ arm was discontinued early due to poor parasite clearance rates by Day 28 (36% Colombia/Suriname & 66% India). The 1 gm AZ arm also showed clearance rates inferior to its comparator arms with day 28 parasite clearance rates of 59% (Colombia/Suriname) and 84% (India). The study conducted with 2 gm of AZ and 600 mg CQ base was a non-comparator study in India and Colombia that showed a parasite clearance rate of 97% (104/107) demonstrating evidence of a dose response relationship. In all three countries, high rates of *P. falciparum* CQ resistance were seen- 92.2% in India and 98.4% in Colombia & Suriname combined [15].

The United States military continues to engage in operations in malaria endemic areas. The current chemoprevention options utilized, while appearing to be effective with only 58 service members diagnosed with malaria in 2018, belie an underlying concern for the future. The 58 infected service members represents a 66% increase over 2017 [16]. In addition, several studies have demonstrated a poor compliance rate with soldiers taking their prophylaxis medications [3, 17–19]. While the data on comparing daily versus weekly administration compliance has been conflicting, weekly administration allows for easier implementation of direct observed therapy (DOT) to ensure compliance [3, 17]. Mefloquine is no longer an option for mass administration in the military and tafenoquine is only now being utilized outside clinical trials. Another weekly chemoprevention agent would have utility within the current options for providers. The re-purposing of AZ which has a known tolerable side effect profile along with CQ, which has been used to treat malaria for decades, presents an inexpensive option that would potentially have a quicker regulatory pathway than a novel, untested compound.

This study aimed to explore the use of the combination of CQ and AZ as a chemoprevention agent for preventing chloroquine resistant *P. falciparum* infection utilizing a human malaria challenge model at the Walter Reed Army Institute of Research (WRAIR). Given the evidence of a possible dose response relationship and the prior success observed at 2 gm, the study utilized a weekly dosing regimen of 2 gm of AZ and 300 mg base of CQ.

## Methods

### Objectives

The primary objective of this study was to assess the safety and efficacy of a weekly CQAZ regimen for prophylaxis against CQ resistant *Plasmodium falciparum*. Secondary objectives were to assess the tolerability and pharmacokinetics of the regimen.

### Study participants

This study was an open-label, randomized controlled trial utilizing a human malaria challenge model. The investigation was conducted at the WRAIR Clinical Trials Center, Silver Spring, MD from 2018 to 2019. Healthy, non-pregnant, non-breastfeeding adults aged 18–50 (inclusive) were eligible for participation. Potentially eligible participants were screened utilizing medical history, physical examination and standard hematologic, renal and liver laboratory evaluations. Laboratory evaluations for human immunodeficiency virus, hepatitis B and C were also conducted. Cardiac risk factors and screening electrocardiogram were assessed. Main exclusion criteria included any chronic medical condition as determined by history, physical examination, or laboratory evaluation that would affect the study results or put the subject at an unacceptably increased risk. Female subjects had to have a negative urine pregnancy test at initial screening and prior to first treatment drug administration and malaria challenge. Women of child bearing potential were required to have been on some form of birth control from 1 month prior to study enrollment and agree to continue at least two forms of birth control for at least 56 days after the challenge. Prior to enrollment, study subjects could not have visited a malaria endemic country in the previous 3 months, received any malaria prophylaxis in the previous 2 months, been diagnosed with malaria within the past 3 years, or have ever received an experimental malaria vaccine. Concomitant medications that could potentially affect the pharmacokinetics of either treatment drug or the prescribed medication, such as cimetidine or other antacids, atorvastatin, or fluconazole or have potential anti-malarial activity were prohibited during the study. The study was registered on ClinicalTrials.gov—NCT03278808 Registered 12 September 2017—Retrospectively registered, https://clinicaltrials.gov/ct2/show/NCT03278808?cond=cq%2Faz&draw=2&rank=1.

### Endpoints

The primary endpoint was symptomatic parasitaemia within 28 days of being challenged. Parasitaemia was determined by microscopic evaluation of thick blood smears. Symptomatic was defined as any one of the following solicited adverse events (AEs) that occurred concurrently with parasitaemia: fever (temperature > 100.4 °F), chills, headache, arthralgia, myalgia, nausea, vomiting, or abdominal pain. Secondary safety endpoints included solicited, unsolicited AEs and electrocardiogram (ECG) findings. Secondary pharmacokinetic endpoints included area under the curve (AUC), maximum concentration (Cmax), time to maximum concentration (Tmax), and half-life (t_½_) for AZ, CQ and chloroquine’s major metabolite, desethylchloroquine (CQm).

### Controlled human malaria infection (CHMI) challenge product

The 7G8 clone was established by Thomas R. Burkot, Department of Entomology, WRAIR in April 1982. It was one of several clones, obtained by limiting dilution, from a Brazilian isolate, IMTM 22. The latter was isolated from a 12-year-old boy near Manaus, Brazil, on 2 March 1980 and cryopreserved. 7G8 was successfully tested for infectivity to mosquitoes in May 1982. It was later tested for drug sensitivity by personnel in the Division of Experimental Therapeutics, WRAIR and was found to be resistant to CQ and susceptible to atovaquone–proguanil. 7G8 has subsequently been used by WRAIR and the Naval Medical Research Center (Silver Spring, MD) for challenges requiring chloroquine resistant *P. falciparum* parasites [20].

### CHMI

The CHMI was conducted at the WRAIR, an institution where over 100 malaria challenge studies have been conducted since 1985. Each subject’s forearm was exposed to five 7G8-infected mosquitoes for a period of 5 min. After this 5-min period, dissection of the mosquito midgut and salivary gland was used to determine if an infectious bite occurred. If none or fewer than 5 infectious mosquitoes bit a subject, then the subject’s forearm was exposed to an additional number of mosquitoes to reach 5 infectious bites total.

### Design

**Fig. 1:**
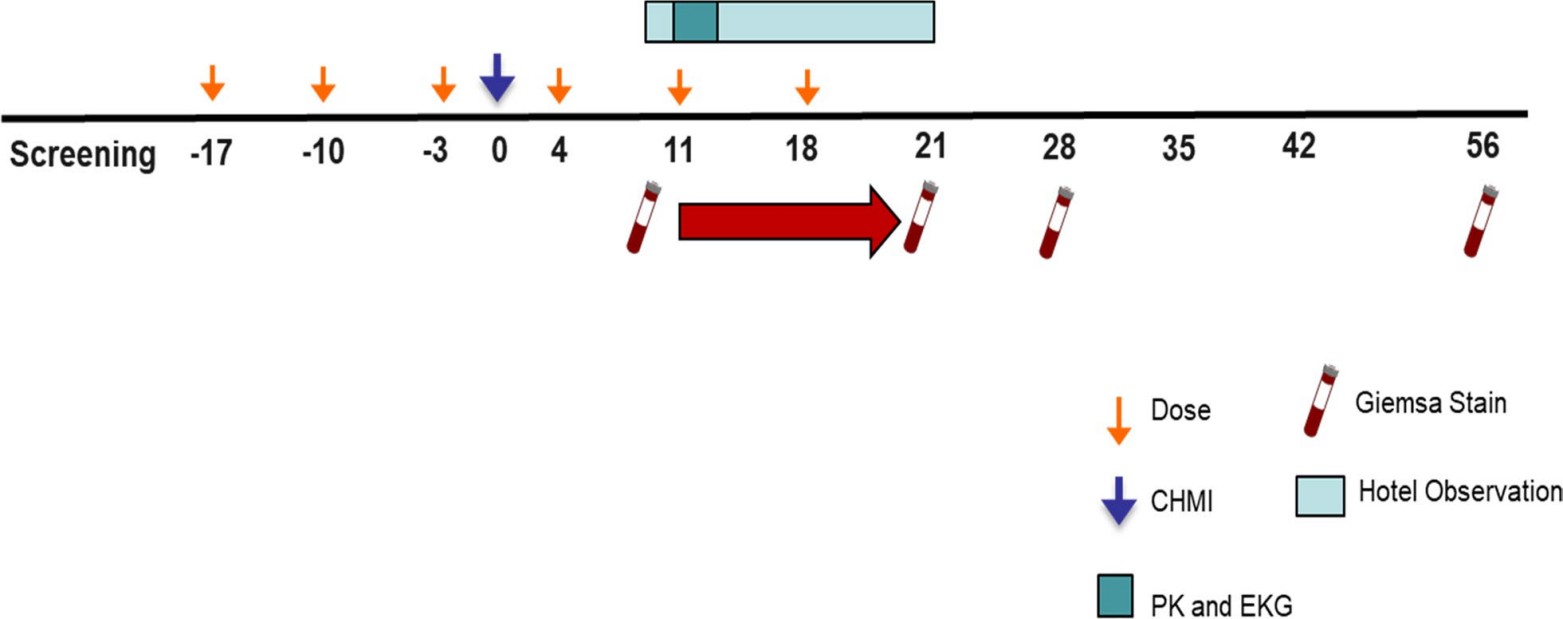
CQAZ study design schema

After enrollment, subjects were randomized into either the CQAZ group or CQ only control group. The CQAZ group received 2 gm of AZ (Greenstone^®^) and 300 mg of CQ base (Natco Pharm Ltd) and the CQ only group received 300 mg of CQ base. Subjects received their treatment medication weekly for 3 weeks prior to the malaria challenge. All treatment medications were administered by study staff and directly observed. Although a standard meal was not provided, all participants were instructed to have eaten prior to taking the CQAZ or CQ, and were provided food if they had not eaten. The challenge occurred midway between weeks 3 and 4 of the study. Eight days after the malaria challenge, subjects checked into and were followed closely at a local hotel with 24-h on-site medical support. Malaria transmission was virtually eliminated by conducting the study during the fall and winter, the small number of subjects, counseling each subject to not leaving the local area and by providing rescue treatment as soon as symptomatic parasitaemia was encountered. Daily Giemsa-stained thick blood film smears for microscopy were obtained on post-challenge days 8–21, in addition to any time a subject reported possible malaria-related symptoms. Microscopists reading the smears were blinded to the treatment group. Parasitaemia was determined using Giemsa-stained blood slides using World Health Organization (WHO)-recommended methods [21]. Subjects that had a positive blood smear for malaria and reported any symptom consistent with possible malaria were considered treatment failures. These subjects stopped receiving weekly CQAZ or CQ and received rescue treatment with 1000 mg atovaquone/400 mg proguanil for 3 days under DOT by study staff. They were then followed until they had 3 consecutive daily negative blood smears. Subjects without symptoms (regardless of smear results) continued to receive weekly CQAZ or CQ for 3 weeks following the challenge. After post challenge day 21, subjects were discharged from the hotel and received follow-up blood smears on day 28 and day 56 and whenever they reported possible malaria-related symptoms (Fig. 1). After the 6th and final dose, any subject who had a positive smear for malaria received rescue treatment with atovaquone/proguanil regardless of whether they were symptomatic, as it was deemed unethical to wait for symptoms given they were no longer scheduled to receive any post-exposure prophylaxis.

Solicited and unsolicited AEs were collected throughout the study as well as haematologic, metabolic, liver and kidney function laboratory evaluations. AEs were graded according to the FDA Toxicity Grading Scale for Healthy Adult and Adolescent Volunteers Enrolled in Preventive Vaccine Clinical Trials. All AEs were coded using the Medical Dictionary for Regulatory Activities version 21.0.

After the 5th weekly dose of CQAZ (3 pre-exposure and 2 post-exposure doses), for any subject who had not yet been deemed a treatment failure, blood samples for drug concentrations were collected at 0, 1, 2, 4, 6, 10, 24, 48, 72, and 96 h post dosing. The same subjects also had an ECG performed approximately 6 h after this dose. QT measurements were corrected using Fridericia’s (QTcF) formulas.

## Bioanalytical methods

### Calibration standard curve and sample preparation

1.00 mg/ml standard stock solutions of CQ, CQm and AZ (US Pharmacopeia, Rockville, MD) were prepared in dimethylsulfoxide (DMSO) and were used to make up a 10 µg/ml mixture of CQ, CQm and AZ in acetonitrile. The calibration standard curve and quality controls (QC) were prepared by spiking blank human plasma (Li-heparin; BioIVT, Westbury, NY) with this 10 µg/ml stock. The calibration standard curve consisted of matrix and an internal standard, mefloquine, with analytes ranging from 0–1000 ng/ml concentrations, with QC samples covering the low, medium, and high concentration ranges of the standard curve. 100 µl of sample was placed in a microcentrifuge tube and 200 µl of acetonitrile with internal standard was added. Each sample was vigorously vortexed for 15 s and centrifuged at 13,000 rpm for 10 min at 4 °C. 220 µl of undisturbed supernatant was transferred to a 96-well plate for liquid chromatography–mass spectrometry (LC–MS) analysis.

The human plasma samples were extracted in the same manner with 200 µl of internal standard added to 100 µl of sample. Concentrations of CQ, CQm and AZ in samples were interpolated from each corresponding standard curve. Samples with concentrations greater than the highest point of the calibration curve were diluted with blank human plasma and extracted in the same manner.

### Liquid chromatography–mass spectrometry (LC-MS/MS) methods

A Waters (Milford, MA) ACQUITY UPLC system was coupled with an AB Sciex (Framingham, MA) QTrap 4000 linear ion trap spectrometer equipped with a Turbo-V source. A Waters CORTECS C18 (2.1 × 50 mm, 2.7 µm) column was maintained at room temperature while the autosampler was maintained at 4 °C to minimize evaporation. Samples were eluted using a linear gradient going from 5% to 95% acetonitrile/0.1% formic acid in water over the course of 1.50 min followed by 1.75 min of isocratic gradient of 95% acetonitrile/0.1% formic acid in water at the flow rate of 0.400 ml/min.

The analysis was performed in multiple reaction monitoring in positive electrospray ionization mode by monitoring the ion transitions from m/z 320.200 → 247.100 (CQ), m/z 292.120 → 114.100 (CQm), m/z 749.601 → 591.400 (AZ), and m/z 379.100 → 361.100 (mefloquine). Compound parameters and source/gas parameters were optimized to obtain the highest intensity of the analytes. The instrument was controlled and data was collected using Analyst^®^ software.

### Pharmacokinetic analysis

The measured plasma concentrations of AZ, CQ and the main chloroquine metabolite (CQm), from each study subject, based on 10 time points per subject, were evaluated. Using the Phoenix WinNonlin 8.1 software (Certara USA, Inc., 100 Overlook Center, Suite 101, Princeton, NJ 08540 USA), non-compartmental analysis (NCA) with the linear up-log down trapezoidal method was performed to calculate the mean pharmacokinetic parameters. Calculated parameters included the t_1/2_, Tmax, Cmax, AUC from 0 to 96 h (AUC_0–96_), extrapolated AUC from 0 to infinity hours (AUC_0–∞_) and the elimination rate constant (Kel).

### Sample size estimate

The desired prophylactic efficacy for anti-malarial drugs in general is defined as approximately 95% compared to placebo. However, given that this was an exploratory proof of concept study efficacy rates > 90% were to be considered a success. The CQ control group was utilized as a measure of malaria challenge success with greater than 1/6 (17%) of subjects not getting study malaria defined as a malaria challenge failure. This number was based on the previous history of malaria challenges at the WRAIR. The study was designed to enroll 12–15 subjects in the CQAZ group with prophylactic success defined as equal to or greater than 91.6% (11/12), 92.3% (12/13), 92.8% (13/14) or 93.3% (14/15).

### Statistical analysis

All reported study data was recorded on the electronic Case Report Forms supplied by Statistics and Data Corporation (SDC) using an Electronic Data Capture clinical database called iMedNet. After data was entered into the clinical study database, electronic edit checks and data review were performed.

Since the measure of success for the primary efficacy analysis was pre-set at greater than 1/12 treatment failures and the CQ group was a control for measuring challenge success, no inferential statistics were performed comparing the efficacy of CQAZ versus CQ. Group characteristics were compared between the two groups using the intention to treat population (ITT). The ITT population was defined as any subject that was enrolled, randomized and received at least one dose of either CQAZ or CQ. Variables were compared using the using Fisher’s Exact Test or two sample T-test. Subjects in the CQAZ group were further analysed based on whether they were considered protected from malaria. Variables in each group (protected (P) vs. non-protected (NP)) were compared using Fisher’s Exact Test or two sample T-test. Pharmacokinetic variables in these two groups were compared using two sample T-test. All inferential tests were performed at the α = 0.05 significance level.

## Results

**Fig. 2:**
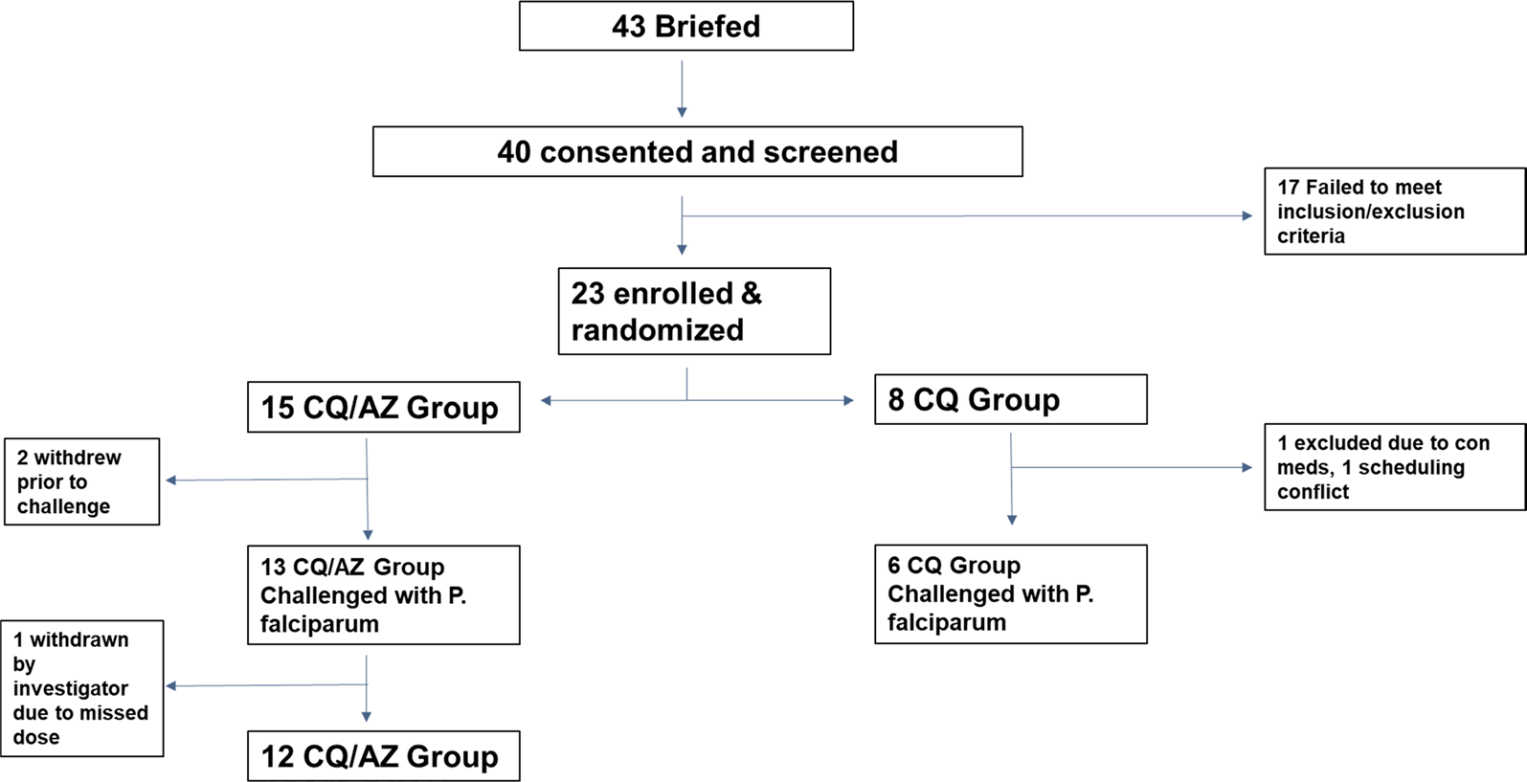
Clinical trial participant flow diagram

Forty (40) potential participants were consented and screened. Twenty-three (23) subjects met all screening criteria and were randomized into the CQAZ (15) or CQ (8) group (Fig. 2). There were no significant differences between the two groups with regards to age, gender, ethnicity or race (Table 1). Two (2) subjects in each group withdrew prior to the malaria challenge. One subject was withdrawn from the CQAZ group after being challenged, due to noncompliance with the study schedule. This subject was followed until the subject had a positive smear and was treated with atovaquone/proguanil. Twelve (12) subjects in the CQAZ and 6 in the CQ group received all 3 doses of weekly CQAZ prophylaxis, were successfully challenged with malaria, and received at least 1 dose of post-exposure prophylaxis.

**Table 1:** Characteristics of ITT clinical trial population

Variable	Chloroquine-azithromycin CQ/AZ (N = 15)	Chloroquine (CQ) (N = 8)	All subjects (N = 23)
Age (years)			
n	15	8	23
Mean (SD)	30.6 (6.38)	33.5 (6.78)	31.6 (6.52)
Median	31.0	35.0	32.0
Min, max	21, 43	23, 41	21, 43
Two sample t-test p-value	0.3209		
Gender: n (%)			
Male	8 (53.3%)	3 (37.5%)	11 (47.8%)
Female	7 (46.7%)	5 (62.5%)	12 (52.2%)
Undifferentiated	0	0	0
Fisher’s exact test p-value	0.6668		
Ethnicity: n(%)			
Hispanic or Latino	1 (6.7%)	1 (12.5%)	(8.7%)
Not hispanic or latino	14 (93.3%)	7 (87.5%)	(91.3%)
Fisher’s exact test p-value	> 0.9999		
Race: n (%)			
American Indian or Alaska	0	0	0
Asian	2 (13.3%)	0	2 (8.7%)
Black or African American	3 (20.0%)	1 (12.5%)	4 (17.4%)
Native Hawaiian or Other Islander	0	0	0
White	9 (60.0%)	5 (62.5%)	(4 60.9%)
Other	0	0	0
Multi-racial	1 (6.7%)	2 (25.0%)	3 (13.0%)
Fisher’s exact test	0.6815		

### Efficacy

In the CQ control group, all 6 subjects had study defined symptomatic malaria while still receiving post-exposure CQ. The mean time to presentation of symptoms with a positive smear was 13 days after the malaria challenge (10–15 days). In the CQAZ cohort, 1/12 subjects presented with symptomatic parasitaemia while still receiving post-exposure CQAZ, on day 9 post-challenge. Two (2) of the remaining 11 subjects had positive smears during the post-exposure prophylaxis period but were asymptomatic. Both of those 2 subjects’ subsequent smears cleared without rescue treatment and they remained in the study. After the 11 remaining subjects received all 6 weekly doses of CQAZ (3 pre-challenge, 3 post-challenge), 6 subjects had positive malaria smears, with 4 of those symptomatic. The mean post-challenge day of presentation for these subjects who had positive smears after finishing all doses of CQAZ was 35 days after challenge (28–41 days). Two subjects who had positive smears on day 28 were asymptomatic and therefore did not meet endpoint criteria but were treated with rescue A/P prior to symptom development. The mean number of days to a positive malaria smear after the last CQAZ dose was 17 days (10–23 days) (Table 2).

**Table 2:** Microscopic parasitemia results

	Chloroquine-Azithromycin CQ/AZ (N = 12)^1^	Chloroquine (CQ) (N = 6)
Parasitemia		
n (%)	7 (58)	6 (100)
Symptomatic parasitemia		
n (%)	5 (42)	6 (100)
Symptomatic parasitemia during post-exposure prophy period^2^		
n (%)	1 (8)	6 (100)
Days to Parasitemia^3^		
Mean (range)	31 (9–41)	13 (10–15)

^**1**^Efficacy population defined as received all 3 doses of pre-exposure prophylaxis, malaria challenged and received at least one dose of post-exposure prophylaxis

^**2**^1-28 days after malaria challenge

^**3**^Days after malaria challenge

Overall, 1/12 in the CQAZ cohort, and 6/6 in the CQ cohort, received 3 weekly doses of either CQ/AZ or CQ, at least 1 post-exposure dose, and met the study definition of malaria (symptomatic for malaria symptoms and a positive blood smear via microscopy) by post-challenge day 28. However, an additional 4 subjects had symptomatic malaria after day 28. Another 2 asymptomatic subjects presented with parasitaemia after the post-exposure treatment period (Day 28) that received rescue treatment and would have likely become symptomatic for malaria.

Further analysis was conducted comparing those in CQAZ group that did not develop parasitaemia, (P) and those that did (NP). Given the low numbers in each group, (P = 5, NP = 7) no statistical differences were found between each group (Table 3).

**Table 3:** Characteristics of CQAZ group based on protection from malaria parasitemia

Variable	Protected (N = 5)	Not protected (N = 7)	All subjects (N = 12)
Age			
n	5	7	12
Mean	27.4	33.7	31.8
Median	31.0	33.0	31.5
Min, max	21, 33	21, 43	21, 43
Two sample t-test	0.1102		
Gender: n (%)			
Male	4 (80.0%)	4 (57.1%)	8 (66.7%)
Female	1 (20.0%)	3 (42.9%)	4 (33.3%)
Fisher’s exact test	0.5758		
Body mass index			
Mean	26.7	30.4	31.1
Median	23.4	29.5	25.3
Two sample t-test	0.4274		
Ethnicity: n (%)			
Hispanic or latino	0	1 (14.2%)	1 (8.3%)
Not hispanic or latino	5 (100.0%)	6 (85.7%)	11 (91.6%)
Fisher’s exact test	> 0.9999		
Race: n (%)			
American Indian or Alaska Native	0	0	0
Asian	0	1 (14.2%)	1 (8.3%)
Black of African American	2 (40.0%)	2 (28.5%)	4 (33.3%)
Native Hawaiian or Other Islander	0	0	0
White	3 (60.0%)	4 (57.1%)	7 (58.3%)
Other	0	0	0
Multi-racial	0	0	0
Fisher’s exact test	0.8674		

Protected group represent CQAZ enrolled subjects that did not have symptomatic parasitemia during post-exposure prophylaxis dosing or a positive smear after dosing completed. The Not Protected group represents CQAZ enrolled subjects with either symptomatic parasitemia during the post-exposure dosing (n = 1) or a positive smear after dosing was completed (n = 6)

### Safety

Both the CQAZ and CQ groups showed high rates of treatment-related AEs, (87% vs 63%) with trends towards higher rates in the CQAZ group (Additional File 1: AE Listings). This trend continued when examining the total number of AEs (35 versus 8) and AEs per person (2.3 versus 1.0). The vast majority of AEs, especially in the CQAZ cohort, were gastrointestinal (GI) related adverse events. Despite the high number of AEs, there were no treatment-related SAEs, and no withdrawals due to treatment related AEs. Only 11% of AEs (5/43) were assessed as being greater than mild in severity. There were 2 AEs judged to be severe. They both occurred in the CQAZ group and were due to nausea. Both AEs lasted less than a day, self-resolved, and the subjects continued in the study. All results, although showing a clear trend towards higher rates in the CQAZ group were not powered to show statistical difference (Table 4). There was no association between subjects who experienced adverse events or severity of adverse events with any pharmacokinetic parameter for AZ, CQ or CQm.

**Table 4:** Safety results

System organ class (SOC)	Chloroquine-azithromycin (CQ/AZ) (N = 15)		Chloroquine (CQ) (N = 8)		All subjects (N = 23)	
	Subjects		Subjects		Subjects	
Preferred term (PT)	Events	n (%)	Events	n (%)	Events	n (%)
Any treatment-related AE p = 0.2969	35	13 (86.7%)	8	5 (62.5%)	43	18 (78.3%)
Gastrointestinal disorders p = 0.0713	28	12 (80.0%)	4	3 (37.5%)	32	15 (65.2%)
Nausea	16	9 (60.0%)	1	1 (12.5%)	17	10 (43.5%)
Diarrhoea	10	8 (53.3%)	1	1 (12.5%)	11	9 (9.1%)
Abdominal pain	1	1 (6.7%)	1	1 (12.5%)	2	2 (8.7%)
Constipation	0	0	1	1 (12.5%)	1	1 (4.3%)
Vomiting	1	1 (6.7%)	0	0	1	1 (4.3%)
General disorders	2	1 (6.7%)	1	1 (12.5%)	3	2 (8.7%)
Fatigue	2	1 (6.7%)	1	1 (12.5%)	3	2 (8.7%)
Infections and infestations	2	2 (13.3%)	1	1 (12.5%)	3	3 (13.0%)
Vulvovaginal mycotic infections	2	2 (13.3%)	1	1 (12.5%)	3	3 (13.0%)
Skin and subcutaneous tissue disorders	3	3 (20.0%)	2	2 (25.0%)	5	5 (21.7%)
Pruritus	3	3 (20.0%)	2	2 (25.0%)	5	5 (21.7%)

The ITT population was used for the safety analysis. Fisher’s exact two-tailed test done

An analysis between the P and NP CQAZ groups showed a trend toward a higher number of AEs in the P group (2.2 vs 1.6 AEs per person). Both the P and NP groups had 1 subject with severe nausea.

An analysis of the laboratory data showed one subject with a potentially relevant clinical abnormality. One subject in the CQAZ had an elevated aspartate aminotransferase (AST) of 126 U/L on Day 28. This subject was found to have a positive malaria smear on this visit. The subject had received all 6 CQAZ doses, with the last dose 10 days prior. While taking CQAZ, the subject’s AST levels had been normal (65 U/l and 72 U/l). Subsequent AST levels normalized and no other liver function test was abnormal. The timing favours malaria infection as the cause of elevated AST rather than the CQAZ. No other subject had any clinically significant haematologic, electrolyte, kidney or liver function laboratory abnormality.

An analysis of the CQAZ group ECG data showed at baseline, a mean QTcF of 406 ms (399–413 ms) (Additional File 2: EKG Data). After the 5th dose of CQAZ, the mean QTcF was 414 ms (394–444 ms). The mean change from baseline was 6.3 ms, with the highest change being 34 ms. Only 1 subject had a change greater than 30 ms. There was no association with this subject and any PK parameter.

### Pharmacokinetic analysis

**Fig. 3:**
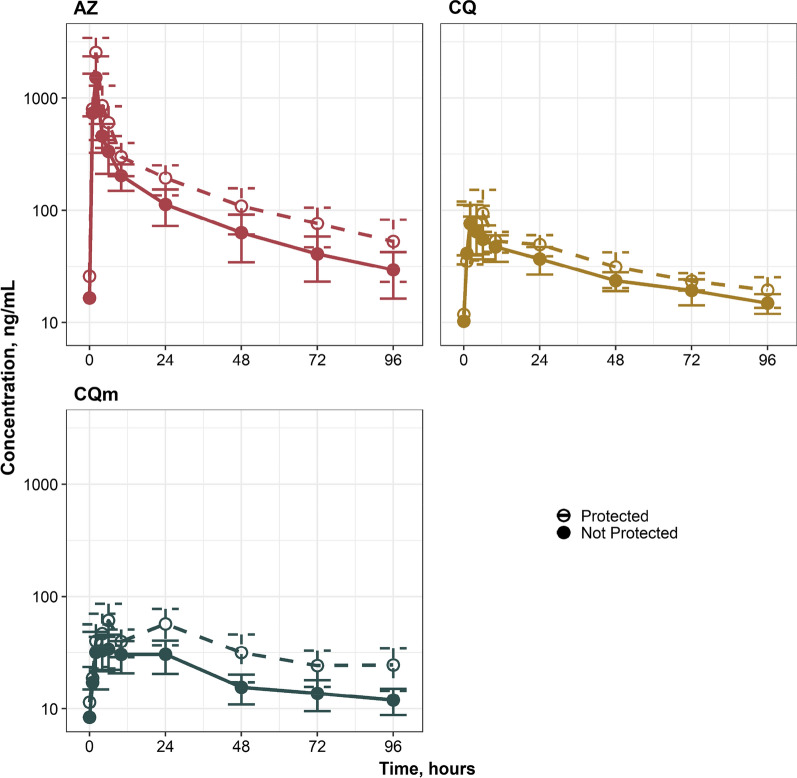
Exposure-time curves for AZ, CQ and CQm in protected and non-protected subgroups of CQAZ group

The pharmacokinetic values of the P and NP groups, as well as overall, are presented in Tables 5, 6 and 7 (Additional file 3: PK Data Points). Pharmacokinetic analysis shows a trend towards higher exposure levels in the P group (Fig. 3). There were significant pharmacokinetic differences in the AZ P and NP groups with the AUC_0–96_ (P-18,946 ng h/ml vs. NP-11,316 ng h/ml, p = 0.044). This trend continued with differences in AUC_0–∞_ (P-22,126 ng h/ml vs. NP- 12,951 ng h/ml p = 0.059) and C_max_ (P-2723 ng/ml vs NP-1682 ng/ml, p = 0.027) showing greater AZ exposure levels over time in the group that did not develop malaria (Table 5).

**Table 5:** Plasma pharmacokinetic parameters of AZ

Parameter	Overall (n = 11)	Infected (n = 6)	Protected (n = 5)	p
*K*_el_ (h^−1^)	0.018 ± 0.0012	0.0183 ± 0.0167	0.02 ± 0.018	0.9
*t*_*1/2*_ (h)	39.36 ± 2.1	38.065 ± 2.88	40.92 ± 3.27	0.53
T_max_ (h)	1.73 ± 0.14	1.67 ± 0.21	1.8 ± 0.2	0.52
C_max_ (ng/ml)	2155.64 ± 250.26	1682.28 ± 283.404	2723.66 ± 275.85	0.027
AUC_0–96_ (ng h/ml)	14,784.64 ± 1783.88	11,316.74 ± 1244.89	18,946.12 ± 2690.78	0.044
AUC_0–∞_ (ng h/ml)	17,122.03 ± 2229.41	12,951.47 ± 1474.19	22,126.71 ± 3549.22	0.059

**Table 6:** Plasma pharmacokinetic parameters of CQ

Parameter	Overall (n = 11)	Infected (n = 6)	Protected (n = 5)	p
*K*_el_ (h^−1^)	0.012 ± 0.0012	0.012 ± 0.0017	0.01 ± 0.012	0.9
*t*_*1/2*_ (h)	65.46 ± 7.48	73.23 ± 12.78	56.14 ± 4.78	0.25
T_max_ (h)	3.36 ± 0.79	3.8 ± 1.33	2.8 ± 0.8	0.52
C_max_ (ng/mL)	94.18 ± 13.95	82.05 ± 14.52	108.74 ± 25.65	0.4
AUC_0–96_ (ng h/mL)	3102.62 ± 241	2760.58 ± 261.9	3513.072 ± 373.27	0.18
AUC_0–∞_ (ng h/mL)	4716.5 ± 388.97	4402.66 ± 531.85	5093.11 ± 585.51	0.41

**Table 7:** Plasma pharmacokinetic parameters of CQm

Parameter	Overall (n = 11)	Infected (n = 6)	Protected (n = 5)	p
*K*_el_ (h^−1^)	0.012 ± 0.0008	0.013 ± 0.00035	0.01 ± 0.0012	0.13
*t*_*1/2*_ (h)	64.55 ± 7.67	53.41 ± 1.49	77.92 ± 15.44	0.19
T_max_ (h)	10.55 ± 2.67	10.67 ± 4.25	10.4 ± 3.49	0.96
C_max_ (ng/mL)	50.25 ± 6.73	38.92 ± 5.41	63.84 ± 10.96	0.088
AUC_0–96_ (ng h/mL)	2582.67 ± 342.62	2821.26 ± 286.52	3394.23 ± 530.82	0.046
AUC_0–∞_ (ng h/mL)	4373.63 ± 767.66	2769.64 ± 345.17	6236.48 ± 1235.93	0.049

Chloroquine and CQm pharmacokinetics also trended towards higher exposure levels in the P group than the NP group, however this difference was not as prominent when compared to their AZ exposure differences (Fig. 3). The biggest difference shown was the AUC of CQm between P and NP group AUC_096_ (P-3394 ng h/ml vs. NP-2821 ng h/ml, p = 0.046) and AUC_0–∞_ (P-6236 ng h/ml vs. NP-2769 ng h/ml, p = 0.049) (Tables 6 and 7).

## Discussion

The evaluated weekly combination of CQAZ was ineffective in preventing malaria infections when given 3 weeks prior to and up to 3 weeks after malaria exposure. Although 92% (11/12) of subjects successfully avoided a symptomatic positive malaria smear while receiving weekly CQAZ, the drug combination was only able to keep the parasite levels to below the microscopic level of detection (typically around 11–50 parasites/ml) while being actively taken [22]. Once the participants stopped taking the CQAZ, the remaining parasites proliferated and 6 more participants had positive malaria smears 1.5 to 3 weeks later. It is unclear whether a longer post-exposure CQAZ course (> 3 weeks) would have resulted in better efficacy. The answer would depend on the mechanism behind the parasite persistence, whether the parasites were arrested in the liver stage or slowly moving through the erythrocyte stage below the level of detection. Only artemisinins have shown the ability to induce *P. falciparum* ring stage dormancy [23]. In addition, in vitro studies have shown that the target of AZ, apicoplasts, are needed for dormant parasites to recover [24]. Regardless, the practical expectation of requiring travelers to be adherent to 4 plus weeks of post-exposure prophylaxis after returning home would likely be unreasonable.

Given that all control CQ subjects had symptomatic parasitaemia within 10-15 days after being challenged, this demonstrated that this was a viable challenge study for testing for prophylaxis of CQ resistant *P. falciparum* malaria.

**Fig. 4:**
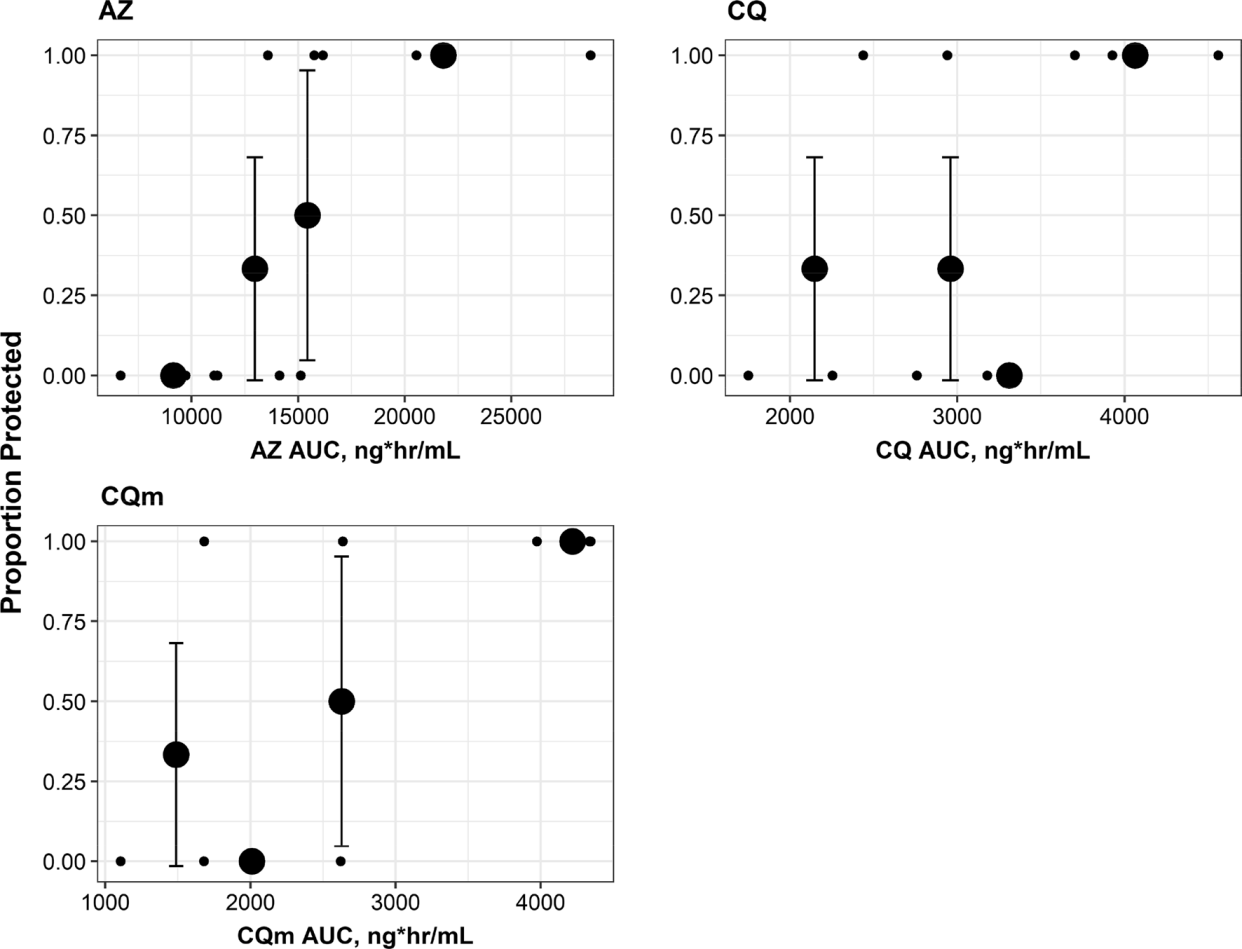
Quantile plots relating exposure of azithromycin, chloroquine and desethylchloroquine to proportion of subjects protected from malaria infection. Solid circles and error bars represent mean AUC of each quantile and the corresponding 80% confidence intervals, respectively

There appeared to be a trend towards higher exposure levels of AZ, CQ and CQm in the group that did not develop parasitaemia during the trial period. Azithromycin demonstrated statistical significance in AUC_0–96_ and Cmax and appeared to have the strongest exposure–response correlation with protection (Fig. 4). The small groups prohibit a strong statistical comparison between the P and NP groups to determine why the P group had higher exposure levels, but there was a trend toward the NP group having higher BMIs. In the study by Kshirsagar et al., findings suggested that body weight inversely affects parasite clearance in those taking AZ [15]. Azithromycin’s high lipophilicity and volume of distribution may have played a role in the differing exposure levels in the NP vs. P groups and subsequent protection.

The overall AE rate, specifically the GI AE rate, was much higher than most studies that have utilized 2 grams of AZ. Studies to treat male urethritis with a single dose of 2 gm showed GI AE rates around 35% [25, 26]. The Zithromax^®^ drug label cites overall GI rates of around 46% with use of 2 gm. The multi-country study by Kshirsagar et al. [15] that tested 2gm of AZ with 600 mg base of CQ reported an overall GI AE rate of 64%. Although the reported rates were higher still, (80% GI AEs), it does suggest that high doses of AZ combined with CQ demonstrate higher GI AE rates than with 2 gm of AZ alone. Despite these high numbers, there were no treatment withdrawals and, similar to the other cited studies, the AEs in this study were primarily mild and transient and required no clinical intervention for resolution. All GI AEs for this study started soon after dosing. Despite the high rates of nausea, only 1 subject experienced emesis. There appeared to be some tolerance of CQAZ as most of the GI AEs in the CQAZ group occurred after one of the first 3 weekly doses (17/28) and only 1 GI AE occurred after dose 4 and 6. However, the highest number of GI AEs occurred after the 5th dose (9/28). From a military perspective, the large-scale use of this particular combination of CQAZ would be impractical if 60–80% of a unit simultaneously suffered GI AEs, no matter how transient. There was no PK association with AE onset or AE severity.

Both CQ and AZ are both associated with QT interval prolongation and this remains a risk with co-administration. The combination of CQAZ has been shown to have a dose dependent but minimal increase in QTc when compared to CQ alone [27]. The CQAZ group in this study did not show any appreciable increase in QTc interval outside of one subject who had an increase of 34 ms but never reached an interval time of clinical concern. Larger studies would need to be done to fully elucidate the QTc and Torsades des Pointes risk of the CQAZ combination.

Most malaria challenge studies have moved to earlier identification of malaria through quantitative polymerase chain reaction (qPCR) to detect and provide rescue treatment before parasite load is high enough for symptoms to develop [28]. This design is most effective when testing a new vaccine or treatment where no subsequent interventions are given once the parasite has been identified. This trial was designed differently as it used symptomatic parasitaemia as the clinical endpoint instead of just the microscopic identification of parasites. This was done to allow for a better replication of real-world practice where travelers, even though bitten by an infected mosquito, would not likely seek medical care unless they were symptomatic and would continue to take their malaria prophylaxis. The post-exposure malaria prophylaxis would then either eradicate the parasite or fail, giving way to parasite replication and symptoms. Given the around the clock monitoring and medical care availability, it was felt that this design would provide an optimal way to show a proof of concept of whether the CQAZ combination of pre and post-exposure prophylaxis would be effective in preventing malaria as it is defined in clinical practice. There were 2 subjects in the CQAZ group who had parasitaemia without symptoms during the post-exposure period. Each subject had subsequent negative malaria smears until 19 days later (35 days post-exposure) when one subject had symptomatic parasitaemia. The other subject remained negative and asymptomatic throughout the remainder of the study.

While the study was powered successfully to determine the efficacy of CQAZ in this experimental model, it was insufficient to meet secondary objective goals. While strong trends existed for safety and pharmacokinetic data, larger numbers of participants would have further clarified these results. This study was a human challenge study that utilized mosquitoes to transmit the malaria rather than direct inoculation of sporozoites through venipuncture. Each method has its scientific merits and drawbacks. One drawback of using mosquitoes is the variable and unquantified number of sporozoites the mosquito injects into each subject [28]. How significant this potential variable played in the results of this study is unclear.

## Conclusion

In conclusion, utilizing a malaria challenge model with chloroquine resistant *P. falciparum* mosquitoes, the weekly combination of CQAZ was not effective in preventing malaria at rates that would be acceptable to justify exploring in further larger scale studies. The GI AE rates from this study would also likely be unacceptable for travellers or a military deployed population.

## Supplementary information

**Additional file 1.** AE Listing: Listing of all adverse events in enrolled subjects.

**Additional file 2.** EKG Data: Lisitng of electrocardiogram values at  baseline and Day 11 after challenge.

**Additional file 3.** PK Data Points: Table of CQ, CQm and AZ exposure levels in the CQAZ cohort.

## Data Availability

The datasets supporting the conclusions of this article are included within the article (and its additional files).

## Abbreviations

AE: Adverse events
AST: Aspartate aminotransferase
AUC: Area under the curve
AUC_0–5_: Area under the curve from 0 to 5 days
AUC_0–∞_: AREA under the curve from 0 to infinity hours
AZ: Azithromycin
BMI: Body Mass Index
Cmax: Maximum concentration
CHMI: Controlled Human Malaria Infection
CQ: Chloroquine
CQAZ: Chloroquine and azithromycin combination
CQm: Desethylchloroquine
DMSO: Dimethylsulfoxide
FDA: US Food and Drug Administration
GI: Gastrointestinal
ITT: Intention to treat population
Kel: Elimination rate constant
LC–MS: Liquid chromatography–mass spectrometry
P: Subjects in CQAZ group protected from malaria
NP: Subjects in CQAZ group not protected from malaria
QC: Quality controls
qPCR: Quantitative polymerase chain reaction
QTcF: Fridericia’s corrected QT interval
T½: Terminal half-life
RNA: Ribonucleic acid
SAE: Serious Adverse Events
SDC: Statistics and Data Corporation
Tmax: Time to maximum concentration
US: United States
WHO: World Health Organization
WRAIR: Walter Reed Army Institute of Research
