# Supplementary material for: An open label study of the safety and efficacy of a single dose of weekly chloroquine and azithromycin administered for malaria prophylaxis in healthy adults challenged with 7G8 chloroquine-resistant Plasmodium falciparum in a controlled human malaria infection model
Source: Malar J. 2020 Sep 16;19:336. doi: 10.1186/s12936-020-03409-z (PMC7493140; doi:10.1186/s12936-020-03409-z)
Supplement: Supplementary file 1 — Additional file 1. AE Listing: Listing of all adverse events in enrolled subjects. [file 12936_2020_3409_MOESM1_ESM.pdf]

Listing 16.2.9.1
Electrocardiogram
All Enrolled Subjects

Subject ID	Actual Treatment Group	Visit (Study Day)/ Date of Collection	HR (bpm)	PR Interval (msec)	QRS Interval (msec)	QT Interval (msec)	QTc Interval (msec)	QT[1] Correction	Overall Results	Describe if Abnormal
2435-041	CQ/AZ	Visit 0 (Day -77 to -18)/ 2018-07-19	55	138	88	400	388	Fridericia	Normal	
		Visit 9 (Day 11)/ 2018-10-15	74	140	100	368	394	Fridericia	Normal	
2435-042	CQ	Visit 0 (Day -77 to -18)/ 2018-07-23	97	158	90	358	420	Fridericia	Normal	
		Visit 9 (Day 11)/ 2018-10-15	N/D	N/D	N/D	N/D	N/D			
2435-043	CQ/AZ	Visit 0 (Day -77 to -18)/ 2018-07-23	45	178	112	452	410	Fridericia	Abnormal (NCS)	sinus bradycardia
2435-044	CQ/AZ	Visit 0 (Day -77 to -18)/ 2018-07-23	54	168	104	430	415	Fridericia	Normal	
		Visit 9 (Day 11)/ 2018-10-15	67	130	112	414	429	Fridericia	Normal	
2435-046	CQ/AZ	Visit 0 (Day -77 to -18)/ 2018-07-24	68	154	78	402	419	Fridericia	Normal	
2435-047	CQ/AZ	Visit 0 (Day -77 to -18)/ 2018-07-24	68	174	106	410	427	Fridericia	Normal	
		Visit 9 (Day 11)/ 2018-10-15	83	184	106	360	401	Fridericia	Normal	

Abbreviations: bpm = Beats Per Minute; CQ/AZ = Chloroquine-Azithromycin; CQ = Chloroquine; CS = Clinically Significant; HR = Heart Rate; NCS = Not Clinically Significant; N/D = Not Done.
Note: CQ/AZ group receives 300 mg of Chloroquine and 2 g of Azithromycin orally weekly. CQ group receives 300 mg of Chloroquine orally weekly.
[1]QTc Interval Calculations: QTcB = QT corrected with Bazett’s formula calculated as QT/[(60/HR)^(1/2)] or QTcF = QT corrected with Fredericia’s formula calculated as QT/[(60/HR)^(1/3)].
Source: SDC-P:\Projects\WRAIR\WRAIR 2435\Statistics\Programs\Primary Programs\TLF\l_eg.sas 30SEP2019 15:15:00

Listing 16.2.9.1
Electrocardiogram
All Enrolled Subjects

Subject ID	Actual Treatment Group	Visit (Study Day)/ Date of Collection	HR (bpm)	PR Interval (msec)	QRS Interval (msec)	QT Interval (msec)	QTc Interval (msec)	QT[1] Correction	Overall Results	Describe if Abnormal
2435-049	CQ/AZ	Visit 0 (Day -77 to -18)/ 2018-07-25	50	142	104	462	434	Fridericia	Normal	
		Visit 9 (Day 11)/ 2018-10-15	58	158	90	428	423	Fridericia	Normal	
2435-051	CQ	Visit 0 (Day -77 to -18)/ 2018-07-26	77	158	94	386	419	Fridericia	Normal	
		Visit 9 (Day 11)/ 2018-10-15	N/D	N/D	N/D	N/D	N/D			
2435-052	CQ/AZ	Visit 0 (Day -77 to -18)/ 2018-07-27	59	152	104	398	395	Fridericia	Normal	
		Visit 9 (Day 11)/ 2018-10-15	70	122	108	376	395	Fridericia	Normal	
2435-055	CQ/AZ	Visit 0 (Day -77 to -18)/ 2018-07-27	59	152	86	388	385	Fridericia	Normal	
		Visit 9 (Day 11)/ 2018-10-15	65	152	100	404	414	Fridericia	Normal	
2435-056	CQ/AZ	Visit 0 (Day -77 to -18)/ 2018-07-30	70	164	84	390	410	Fridericia	Normal	
		Visit 9 (Day 11)/ 2018-10-15	81	162	96	402	444	Fridericia	Normal	

Abbreviations: bpm = Beats Per Minute; CQ/AZ = Chloroquine-Azithromycin; CQ = Chloroquine; CS = Clinically Significant; HR = Heart Rate; NCS = Not Clinically Significant; N/D = Not Done.
Note: CQ/AZ group receives 300 mg of Chloroquine and 2 g of Azithromycin orally weekly. CQ group receives 300 mg of Chloroquine orally weekly.
[1]QTc Interval Calculations: QTcB = QT corrected with Bazett’s formula calculated as QT/[(60/HR)^(1/2)] or QTcF = QT corrected with Fredericia’s formula calculated as QT/[(60/HR)^(1/3)].
Source: SDC-P:\Projects\WRAIR\WRAIR 2435\Statistics\Programs\Primary Programs\TLF\l_eg.sas 30SEP2019 15:15:00

Listing 16.2.9.1
Electrocardiogram
All Enrolled Subjects

Subject ID	Actual Treatment Group	Visit (Study Day)/ Date of Collection	HR (bpm)	PR Interval (msec)	QRS Interval (msec)	QT Interval (msec)	QTc Interval (msec)	QT[1] Correction	Overall Results	Describe if Abnormal
2435-057	CQ	Visit 0 (Day -77 to -18)/ 2018-08-01	68	122	88	430	448	Fridericia	Normal	
		Visit 9 (Day 11)/ 2018-10-15	N/D	N/D	N/D	N/D	N/D			
2435-059	CQ/AZ	Visit 0 (Day -77 to -18)/ 2018-08-07	51	182	102	436	413	Fridericia	Normal	
		Visit 9 (Day 11)/ 2018-10-15	55	172	100	426	413	Fridericia	Normal	
		Unscheduled/ 2018-11-13	102	148	106	338	403	Fridericia	Abnormal (NCS)	sinus tachycardia
2435-060	CQ	Visit 0 (Day -77 to -18)/ 2018-08-07	71	194	110	400	423	Fridericia	Normal	
		Visit 9 (Day 11)/ 2018-10-15	N/D	N/D	N/D	N/D	N/D			
2435-067	CQ	Visit 0 (Day -77 to -18)/ 2018-08-21	65	164	84	434	445	Fridericia	Normal	
		Visit 9 (Day 11)/ 2018-10-15	N/D	N/D	N/D	N/D	N/D			

Abbreviations: bpm = Beats Per Minute; CQ/AZ = Chloroquine-Azithromycin; CQ = Chloroquine; CS = Clinically Significant; HR = Heart Rate; NCS = Not Clinically Significant; N/D = Not Done.
Note: CQ/AZ group receives 300 mg of Chloroquine and 2 g of Azithromycin orally weekly. CQ group receives 300 mg of Chloroquine orally weekly.
[1]QTc Interval Calculations: QTcB = QT corrected with Bazett’s formula calculated as QT/[(60/HR)^(1/2)] or QTcF = QT corrected with Fredericia’s formula calculated as QT/[(60/HR)^(1/3)].
Source: SDC-P:\Projects\WRAIR\WRAIR 2435\Statistics\Programs\Primary Programs\TLF\l_eg.sas 30SEP2019 15:15:00

Listing 16.2.9.1
Electrocardiogram
All Enrolled Subjects

Subject ID	Actual Treatment Group	Visit (Study Day)/ Date of Collection	HR (bpm)	PR Interval (msec)	QRS Interval (msec)	QT Interval (msec)	QTc Interval (msec)	QT[1] Correction	Overall Results	Describe if Abnormal
2435-068	CQ/AZ	Visit 0 (Day -77 to -18)/ 2018-08-21	53	158	82	402	385	Fridericia	Normal	
2435-070	CQ	Visit 0 (Day -77 to -18)/ 2018-08-23	72	150	90	380	403	Fridericia	Normal	
2435-071	CQ	Visit 0 (Day -77 to -18)/ 2018-08-23	66	160	90	396	408	Fridericia	Normal	
2435-074	CQ	Visit 0 (Day -77 to -18)/ 2018-08-24	73	180	98	412	439	Fridericia	Normal	
		Visit 9 (Day 11)/ 2018-10-15	N/D	N/D	N/D	N/D	N/D			
2435-075	CQ/AZ	Visit 0 (Day -77 to -18)/ 2018-08-27	58	162	102	400	395	Fridericia	Normal	
		Visit 9 (Day 11)/ 2018-10-15	65	164	94	390	400	Fridericia	Normal	
2435-077	CQ/AZ	Visit 0 (Day -77 to -18)/ 2018-08-27	66	120	82	404	417	Fridericia	Normal	
		Visit 9 (Day 11)/ 2018-10-15	73	132	86	394	420	Fridericia	Normal	

Abbreviations: bpm = Beats Per Minute; CQ/AZ = Chloroquine-Azithromycin; CQ = Chloroquine; CS = Clinically Significant; HR = Heart Rate; NCS = Not Clinically Significant; N/D = Not Done.
Note: CQ/AZ group receives 300 mg of Chloroquine and 2 g of Azithromycin orally weekly. CQ group receives 300 mg of Chloroquine orally weekly.
[1]QTc Interval Calculations: QTcB = QT corrected with Bazett's formula calculated as QT/[(60/HR)^(1/2)] or QTcF = QT corrected with Fredericia's formula calculated as QT/[(60/HR)^(1/3)].
Source: SDC-P:\Projects\WRAIR\WRAIR 2435\Statistics\Programs\Primary Programs\TLF\l_eg.sas 30SEP2019 15:15:00

Listing 16.2.9.1
Electrocardiogram
All Enrolled Subjects

Subject ID	Actual Treatment Group	Visit (Study Day)/ Date of Collection	HR (bpm)	PR Interval (msec)	QRS Interval (msec)	QT Interval (msec)	QTc Interval (msec)	QT[1] Correction	Overall Results	Describe if Abnormal
2435-078	CQ/AZ	Visit 0 (Day -77 to -18)/ 2018-08-28	68	172	98	376	392	Fridericia	Normal	
		Visit 9 (Day 11)/ 2018-10-15	N/D	N/D	N/D	N/D	N/D			
2435-080	CQ/AZ	Visit 0 (Day -77 to -18)/ 2018-08-30	67	124	86	392	406	Fridericia	Normal	
		Visit 9 (Day 11)/ 2018-10-15	86	180	53	374	421	Fridericia	Normal	

Abbreviations: bpm = Beats Per Minute; CQ/AZ = Chloroquine-Azithromycin; CQ = Chloroquine; CS = Clinically Significant; HR = Heart Rate; NCS = Not Clinically Significant; N/D = Not Done.
Note: CQ/AZ group receives 300 mg of Chloroquine and 2 g of Azithromycin orally weekly. CQ group receives 300 mg of Chloroquine orally weekly.
[1]QTc Interval Calculations: QTcB = QT corrected with Bazett’s formula calculated as QT/[(60/HR)^(1/2)] or QTcF = QT corrected with Fredericia’s formula calculated as QT/[(60/HR)^(1/3)].
Source: SDC-P:\Projects\WRAIR\WRAIR 2435\Statistics\Programs\Primary Programs\TLF\l_eg.sas 30SEP2019 15:15:00
