# Supplementary material for: An open label study of the safety and efficacy of a single dose of weekly chloroquine and azithromycin administered for malaria prophylaxis in healthy adults challenged with 7G8 chloroquine-resistant Plasmodium falciparum in a controlled human malaria infection model
Source: Malar J. 2020 Sep 16;19:336. doi: 10.1186/s12936-020-03409-z (PMC7493140; doi:10.1186/s12936-020-03409-z)
Supplement: Supplementary file 2 — Additional file 2. EKG Data: Lisitng of electrocardiogram values at baseline and Day 11 after challenge. [file 12936_2020_3409_MOESM2_ESM.pdf]

Listing 16.2.7.1
All Adverse Events
All Enrolled Subjects

Subject ID	Actual Treatment/ Solicited?	Adverse Event/ System Organ Class/ Preferred Term	Onset Date/ Resolution Date or Ongoing	Was Event Serious?	Reason for Seriousness[1]	Severity	Relationship to Study Drug	Action Taken with Study Drug	Outcome[2]
2435-041	CQ/AZ/ Yes	ARTHRALGIA/ Musculoskeletal and connective tissue disorders/ Arthralgia	2018-10-12/ 2018-10-13	No		Mild	Unlikely Related	Dose Not Changed	3
	CQ/AZ/ Yes	ARTHRALGIA/ Musculoskeletal and connective tissue disorders/ Arthralgia	2018-10-18/ 2018-10-18	No		Mild	Not Related	Dose Not Changed	3
	CQ/AZ/ Yes	DIZZINESS/ Nervous system disorders/ Dizziness	2018-10-15/ 2018-10-15	No		Mild	Unlikely Related	Dose Not Changed	3
	CQ/AZ/ Yes	DIZZINESS/ Nervous system disorders/ Dizziness	2018-10-18/ 2018-10-18	No		Mild	Not Related	Dose Not Changed	3
	CQ/AZ/ Yes	FATIGUE/ General disorders and administration site conditions/ Fatigue	2018-10-01/ 2018-10-01	No		Mild	Probably Related	Dose Not Changed	3
	CQ/AZ/ Yes	FATIGUE/ General disorders and administration site conditions/ Fatigue	2018-10-05/ 2018-10-08	No		Mild	Probably Related	Dose Not Changed	3

Abbreviations: CQ/AZ = Chloroquine-Azithromycin; CQ = Chloroquine.
Note: CQ/AZ group receives 300 mg of Chloroquine and 2 g of Azithromycin orally weekly. CQ group receives 300 mg of Chloroquine orally weekly. Adverse Events are coded using Medical Dictionary for Regulatory Activities Version 21.1.
[1] 1 = Death; 2 = Life-Threatening; 3 = Requires inpatient hospitalization or prolongation of existing hospitalization; 4 = Results in persistent or significant disability/incapacity; 5 = Congenital anomaly/birth defect; 6 = Other medically important event.
[2] 1 = Fatal; 2 = Not Recovered/Not Resolved; 3 = Recovered/Resolved; 4 = Recovered/Resolved with Sequelae; 5 = Recovering/Resolving; 6 = Unknown.
Source: SDC-P:\Projects\WRAIR\WRAIR 2435\Statistics\Programs\Primary Programs\TLF\l_ae.sas 01OCT2019 14:05:50

Listing 16.2.7.1
All Adverse Events
All Enrolled Subjects

Subject ID	Actual Treatment/ Solicited?	Adverse Event/ System Organ Class/ Preferred Term	Onset Date/ Resolution Date or Ongoing	Was Event Serious?	Reason for Serious-ness[1]	Severity	Relationship to Study Drug	Action Taken with Study Drug	Outcome[2]
	CQ/AZ/ Yes	FATIGUE/ General disorders and administration site conditions/ Fatigue	2018-10-12/ 2018-10-12	No		Mild	Unlikely Related	Dose Not Changed	3
	CQ/AZ/ Yes	FATIGUE/ General disorders and administration site conditions/ Fatigue	2018-10-16/ 2018-10-16	No		Mild	Unlikely Related	Dose Not Changed	3

Abbreviations: CQ/AZ = Chloroquine-Azithromycin; CQ = Chloroquine.
Note: CQ/AZ group receives 300 mg of Chloroquine and 2 g of Azithromycin orally weekly. CQ group receives 300 mg of Chloroquine orally weekly. Adverse Events are coded using Medical Dictionary for Regulatory Activities Version 21.1.
[1] 1 = Death; 2 = Life-Threatening; 3 = Requires inpatient hospitalization or prolongation of existing hospitalization; 4 = Results in persistent or significant disability/incapacity; 5 = Congenital anomaly/birth defect; 6 = Other medically important event.
[2] 1 = Fatal; 2 = Not Recovered/Not Resolved; 3 = Recovered/Resolved; 4 = Recovered/Resolved with Sequelae; 5 = Recovering/Resolving; 6 = Unknown.
Source: SDC-P:\Projects\WRAIR\WRAIR 2435\Statistics\Programs\Primary Programs\TLF\l_ae.sas 01OCT2019 14:05:50

Listing 16.2.7.1
All Adverse Events
All Enrolled Subjects

Subject ID	Actual Treatment/ Solicited?	Adverse Event/ System Organ Class/ Preferred Term	Onset Date/ Resolution Date or Ongoing	Was Event Serious?	Reason for Seriousness[1]	Severity	Relationship to Study Drug	Action Taken with Study Drug	Outcome[2]
2435-041	CQ/AZ/ Yes	FATIGUE/ General disorders and administration site conditions/ Fatigue	2018-10-18/ 2018-10-18	No		Mild	Not Related	Dose Not Changed	3
	CQ/AZ/ Yes	MYALGIA/ Musculoskeletal and connective tissue disorders/ Myalgia	2018-10-18/ 2018-10-18	No		Mild	Not Related	Dose Not Changed	3
	CQ/AZ/ No	Malaria/ Infections and infestations/ Malaria	2018-11-14/ 2018-11-16	No		Mild	Possibly Related	Not Applicable	3
	CQ/AZ/ Yes	PRURITUS/ Skin and subcutaneous tissue disorders/ Pruritus	2018-10-18/ 2018-10-18	No		Mild	Not Related	Dose Not Changed	3
	CQ/AZ/ No	Schizophrenia/ Psychiatric disorders/ Schizophrenia	2018-11-17/ 2019-03-18	Yes	6	Severe	Unlikely Related	Not Applicable	4
2435-042	CQ/ No	Allergic Reaction to Ma/ Immune system disorders/ Drug hypersensitivity	2018-10-19/ 2018-10-22	No		Moderate	Not Related	Not Applicable	3
	CQ/ No	Local Hypersensitivity/ Immune system disorders/ Allergy to arthropod bite	2018-10-05/ 2018-10-08	No		Moderate	Not Related	Dose Not Changed	3

Abbreviations: CQ/AZ = Chloroquine-Azithromycin; CQ = Chloroquine.
Note: CQ/AZ group receives 300 mg of Chloroquine and 2 g of Azithromycin orally weekly. CQ group receives 300 mg of Chloroquine orally weekly. Adverse Events are coded using Medical Dictionary for Regulatory Activities Version 21.1.
[1] 1 = Death; 2 = Life-Threatening; 3 = Requires inpatient hospitalization or prolongation of existing hospitalization; 4 = Results in persistent or significant disability/incapacity; 5 = Congenital anomaly/birth defect; 6 = Other medically important event.
[2] 1 = Fatal; 2 = Not Recovered/Not Resolved; 3 = Recovered/Resolved; 4 = Recovered/Resolved with Sequelae; 5 = Recovering/Resolving; 6 = Unknown.
Source: SDC-P:\Projects\WRAIR\WRAIR 2435\Statistics\Programs\Primary Programs\TLF\l_ae.sas 01OCT2019 14:05:50

Listing 16.2.7.1
All Adverse Events
All Enrolled Subjects

Subject ID	Actual Treatment/ Solicited?	Adverse Event/ System Organ Class/ Preferred Term	Onset Date/ Resolution Date or Ongoing	Was Event Serious?	Reason for Serious-ness[1]	Severity	Relationship to Study Drug	Action Taken with Study Drug	Outcome[2]
	CQ/ No	Malaria/ Infections and infestations/ Malaria	2018-10-18/ 2018-10-22	No		Moderate	Unlikely Related	Not Applicable	3
	CQ/ Yes	PRURITUS/ Skin and subcutaneous tissue disorders/ Pruritus	2018-10-04/ 2018-10-08	No		Mild	Not Related	Dose Not Changed	3

Abbreviations: CQ/AZ = Chloroquine-Azithromycin; CQ = Chloroquine.
Note: CQ/AZ group receives 300 mg of Chloroquine and 2 g of Azithromycin orally weekly. CQ group receives 300 mg of Chloroquine orally weekly. Adverse Events are coded using Medical Dictionary for Regulatory Activities Version 21.1.
[1] 1 = Death; 2 = Life-Threatening; 3 = Requires inpatient hospitalization or prolongation of existing hospitalization; 4 = Results in persistent or significant disability/incapacity; 5 = Congenital anomaly/birth defect; 6 = Other medically important event.
[2] 1 = Fatal; 2 = Not Recovered/Not Resolved; 3 = Recovered/Resolved; 4 = Recovered/Resolved with Sequelae; 5 = Recovering/Resolving; 6 = Unknown.
Source: SDC-P:\Projects\WRAIR\WRAIR 2435\Statistics\Programs\Primary Programs\TLF\l_ae.sas 01OCT2019 14:05:50

Listing 16.2.7.1
All Adverse Events
All Enrolled Subjects

Subject ID	Actual Treatment/ Solicited?	Adverse Event/ System Organ Class/ Preferred Term	Onset Date/ Resolution Date or Ongoing	Was Event Serious?	Reason for Seriousness[1]	Severity	Relationship to Study Drug	Action Taken with Study Drug	Outcome[2]
2435-042	CQ/ Yes	VAGINAL YEAST INFECTION/ Infections and infestations/ Vulvovaginal mycotic infection	2018-09-26/ 2018-10-04	No		Mild	Unlikely Related	Dose Not Changed	3
2435-044	CQ/AZ/ Yes	MYALGIA/ Musculoskeletal and connective tissue disorders/ Myalgia	2018-10-21/ 2018-10-22	No		Mild	Not Related	Dose Not Changed	3
	CQ/AZ/ No	Malaria/ Infections and infestations/ Malaria	2018-11-29/ 2018-11-30	No		Mild	Not Related	Not Applicable	3
	CQ/AZ/ Yes	NAUSEA/ Gastrointestinal disorders/ Nausea	2018-09-24/ 2018-09-24	No		Mild	Probably Related	Dose Not Changed	3
	CQ/AZ/ Yes	NAUSEA/ Gastrointestinal disorders/ Nausea	2018-10-08/ 2018-10-08	No		Moderate	Definitely Related	Dose Not Changed	3
	CQ/AZ/ Yes	NAUSEA/ Gastrointestinal disorders/ Nausea	2018-10-15/ 2018-10-15	No		Moderate	Definitely Related	Dose Not Changed	3

Abbreviations: CQ/AZ = Chloroquine-Azithromycin; CQ = Chloroquine.
Note: CQ/AZ group receives 300 mg of Chloroquine and 2 g of Azithromycin orally weekly. CQ group receives 300 mg of Chloroquine orally weekly. Adverse Events are coded using Medical Dictionary for Regulatory Activities Version 21.1.
[1] 1 = Death; 2 = Life-Threatening; 3 = Requires inpatient hospitalization or prolongation of existing hospitalization; 4 = Results in persistent or significant disability/incapacity; 5 = Congenital anomaly/birth defect; 6 = Other medically important event.
[2] 1 = Fatal; 2 = Not Recovered/Not Resolved; 3 = Recovered/Resolved; 4 = Recovered/Resolved with Sequelae; 5 = Recovering/Resolving; 6 = Unknown.
Source: SDC-P:\Projects\WRAIR\WRAIR 2435\Statistics\Programs\Primary Programs\TLF\l_ae.sas 01OCT2019 14:05:50

Listing 16.2.7.1
All Adverse Events
All Enrolled Subjects

Subject ID	Actual Treatment/ Solicited?	Adverse Event/ System Organ Class/ Preferred Term	Onset Date/ Resolution Date or Ongoing	Was Event Serious?	Reason for Serious-ness[1]	Severity	Relationship to Study Drug	Action Taken with Study Drug	Outcome[2]
2435-046	CQ/AZ/ Yes	DIARRHEA/ Gastrointestinal disorders/ Diarrhoea	2018-09-17/ 2018-09-17	No		Mild	Definitely Related	Dose Not Changed	3
	CQ/AZ/ Yes	DIARRHEA/ Gastrointestinal disorders/ Diarrhoea	2018-09-24/ 2018-09-24	No		Mild	Definitely Related	Dose Not Changed	3

Abbreviations: CQ/AZ = Chloroquine-Azithromycin; CQ = Chloroquine.
Note: CQ/AZ group receives 300 mg of Chloroquine and 2 g of Azithromycin orally weekly. CQ group receives 300 mg of Chloroquine orally weekly. Adverse Events are coded using Medical Dictionary for Regulatory Activities Version 21.1.
[1]1 = Death; 2 = Life-Threatening; 3 = Requires inpatient hospitalization or prolongation of existing hospitalization; 4 = Results in persistent or significant disability/incapacity; 5 = Congenital anomaly/birth defect; 6 = Other medically important event.
[2]1 = Fatal; 2 = Not Recovered/Not Resolved; 3 = Recovered/Resolved; 4 = Recovered/Resolved with Sequelae; 5 = Recovering/Resolving; 6 = Unknown.
Source: SDC-P:\Projects\WRAIR\WRAIR 2435\Statistics\Programs\Primary Programs\TLF\l_ae.sas 01OCT2019 14:05:50

Listing 16.2.7.1
All Adverse Events
All Enrolled Subjects

Subject ID	Actual Treatment/ Solicited?	Adverse Event/ System Organ Class/ Preferred Term	Onset Date/ Resolution Date or Ongoing	Was Event Serious?	Reason for Seriousness[1]	Severity	Relationship to Study Drug	Action Taken with Study Drug	Outcome[2]
2435-046	CQ/AZ/ No	Malaria/ Infections and infestations/ Malaria	2018-10-13/ 2018-10-17	No		Mild	Unlikely Related	Not Applicable	3
	CQ/AZ/ Yes	NAUSEA/ Gastrointestinal disorders/ Nausea	2018-09-17/ 2018-09-17	No		Severe	Definitely Related	Dose Not Changed	3
	CQ/AZ/ Yes	NAUSEA/ Gastrointestinal disorders/ Nausea	2018-09-24/ 2018-09-24	No		Severe	Definitely Related	Dose Not Changed	3
	CQ/AZ/ Yes	PRURITUS/ Skin and subcutaneous tissue disorders/ Pruritus	2018-10-04/ 2018-10-18	No		Mild	Definitely Related	Dose Not Changed	3
	CQ/AZ/ No	Right shoulder pain/ Musculoskeletal and connective tissue disorders/ Musculoskeletal pain	2018-10-03/ 2018-10-09	No		Mild	Not Related	Dose Not Changed	3
	CQ/AZ/ Yes	VAGINAL YEAST INFECTION/ Infections and infestations/ Vulvovaginal mycotic infection	2018-10-16/ 2018-10-22	No		Mild	Probably Related	Not Applicable	3
	CQ/AZ/ No	post Abdominoplasty pai/ Injury, poisoning and procedural complications/ Procedural pain	2019-03-04/ ONGOING	No		Moderate	Not Related	Not Applicable	5

Abbreviations: CQ/AZ = Chloroquine-Azithromycin; CQ = Chloroquine.
Note: CQ/AZ group receives 300 mg of Chloroquine and 2 g of Azithromycin orally weekly. CQ group receives 300 mg of Chloroquine orally weekly. Adverse Events are coded using Medical Dictionary for Regulatory Activities Version 21.1.
[1] 1 = Death; 2 = Life-Threatening; 3 = Requires inpatient hospitalization or prolongation of existing hospitalization; 4 = Results in persistent or significant disability/incapacity; 5 = Congenital anomaly/birth defect; 6 = Other medically important event.
[2] 1 = Fatal; 2 = Not Recovered/Not Resolved; 3 = Recovered/Resolved; 4 = Recovered/Resolved with Sequelae; 5 = Recovering/Resolving; 6 = Unknown.
Source: SDC-P:\Projects\WRAIR\WRAIR 2435\Statistics\Programs\Primary Programs\TLF\l_ae.sas 01OCT2019 14:05:50

Listing 16.2.7.1
All Adverse Events
All Enrolled Subjects

Subject ID	Actual Treatment/ Solicited?	Adverse Event/ System Organ Class/ Preferred Term	Onset Date/ Resolution Date or Ongoing	Was Event Serious?	Reason for Seriousness[1]	Severity	Relationship to Study Drug	Action Taken with Study Drug	Outcome[2]
2435-047	CQ/AZ/ No	Malaria/ Infections and infestations/ Malaria	2018-11-09/ 2018-11-11	No		Mild	Unlikely Related	Not Applicable	3
	CQ/AZ/ Yes	NAUSEA/ Gastrointestinal disorders/ Nausea	2018-10-15/ 2018-10-15	No		Mild	Probably Related	Dose Not Changed	3

Abbreviations: CQ/AZ = Chloroquine-Azithromycin; CQ = Chloroquine.
Note: CQ/AZ group receives 300 mg of Chloroquine and 2 g of Azithromycin orally weekly. CQ group receives 300 mg of Chloroquine orally weekly. Adverse Events are coded using Medical Dictionary for Regulatory Activities Version 21.1.
[1] 1 = Death; 2 = Life-Threatening; 3 = Requires inpatient hospitalization or prolongation of existing hospitalization; 4 = Results in persistent or significant disability/incapacity; 5 = Congenital anomaly/birth defect; 6 = Other medically important event.
[2] 1 = Fatal; 2 = Not Recovered/Not Resolved; 3 = Recovered/Resolved; 4 = Recovered/Resolved with Sequelae; 5 = Recovering/Resolving; 6 = Unknown.
Source: SDC-P:\Projects\WRAIR\WRAIR 2435\Statistics\Programs\Primary Programs\TLF\l_ae.sas 01OCT2019 14:05:50

Listing 16.2.7.1
All Adverse Events
All Enrolled Subjects

Subject ID	Actual Treatment/ Solicited?	Adverse Event/ System Organ Class/ Preferred Term	Onset Date/ Resolution Date or Ongoing	Was Event Serious?	Reason for Seriousness[1]	Severity	Relationship to Study Drug	Action Taken with Study Drug	Outcome[2]
2435-049	CQ/AZ/ Yes	DIARRHEA/ Gastrointestinal disorders/ Diarrhoea	2018-10-01/ 2018-10-01	No		Mild	Definitely Related	Dose Not Changed	3
	CQ/AZ/ No	Elevated Aspartate Amin/ Investigations/ Aspartate aminotransferase increased	2018-11-01/ ONGOING	No		Moderate	Unlikely Related	Dose Not Changed	5
	CQ/AZ/ No	Malaria/ Infections and infestations/ Malaria	2018-11-01/ 2018-11-07	No		Mild	Unlikely Related	Dose Not Changed	3
	CQ/AZ/ Yes	NAUSEA/ Gastrointestinal disorders/ Nausea	2018-09-24/ 2018-09-24	No		Severe	Probably Related	Dose Not Changed	3
	CQ/AZ/ Yes	NAUSEA/ Gastrointestinal disorders/ Nausea	2018-10-01/ 2018-10-01	No		Severe	Definitely Related	Dose Not Changed	3
	CQ/AZ/ Yes	NAUSEA/ Gastrointestinal disorders/ Nausea	2018-10-15/ 2018-10-15	No		Mild	Probably Related	Dose Not Changed	3
	CQ/AZ/ Yes	PRURITUS/ Skin and subcutaneous tissue disorders/ Pruritus	2018-10-04/ 2018-10-13	No		Mild	Definitely Related	Dose Not Changed	3

Abbreviations: CQ/AZ = Chloroquine-Azithromycin; CQ = Chloroquine.
Note: CQ/AZ group receives 300 mg of Chloroquine and 2 g of Azithromycin orally weekly. CQ group receives 300 mg of Chloroquine orally weekly. Adverse Events are coded using Medical Dictionary for Regulatory Activities Version 21.1.
[1] 1 = Death; 2 = Life-Threatening; 3 = Requires inpatient hospitalization or prolongation of existing hospitalization; 4 = Results in persistent or significant disability/incapacity; 5 = Congenital anomaly/birth defect; 6 = Other medically important event.
[2] 1 = Fatal; 2 = Not Recovered/Not Resolved; 3 = Recovered/Resolved; 4 = Recovered/Resolved with Sequelae; 5 = Recovering/Resolving; 6 = Unknown.
Source: SDC-P:\Projects\WRAIR\WRAIR 2435\Statistics\Programs\Primary Programs\TLF\l_ae.sas 01OCT2019 14:05:50

Listing 16.2.7.1
All Adverse Events
All Enrolled Subjects

Subject ID	Actual Treatment/ Solicited?	Adverse Event/ System Organ Class/ Preferred Term	Onset Date/ Resolution Date or Ongoing	Was Event Serious?	Reason for Serious-ness[1]	Severity	Relationship to Study Drug	Action Taken with Study Drug	Outcome[2]
	CQ/AZ/ No	rash on right forearm d/ Injury, poisoning and procedural complications/ Arthropod bite	2018-10-04/ 2018-10-16	No		Mild	Not Related	Dose Not Changed	3
2435-051	CQ/ No	Malaria/ Infections and infestations/ Malaria	2018-10-16/ 2018-10-19	No		Moderate	Unlikely Related	Not Applicable	3

Abbreviations: CQ/AZ = Chloroquine-Azithromycin; CQ = Chloroquine.
Note: CQ/AZ group receives 300 mg of Chloroquine and 2 g of Azithromycin orally weekly. CQ group receives 300 mg of Chloroquine orally weekly. Adverse Events are coded using Medical Dictionary for Regulatory Activities Version 21.1.
[1] 1 = Death; 2 = Life-Threatening; 3 = Requires inpatient hospitalization or prolongation of existing hospitalization; 4 = Results in persistent or significant disability/incapacity; 5 = Congenital anomaly/birth defect; 6 = Other medically important event.
[2] 1 = Fatal; 2 = Not Recovered/Not Resolved; 3 = Recovered/Resolved; 4 = Recovered/Resolved with Sequelae; 5 = Recovering/Resolving; 6 = Unknown.
Source: SDC-P:\Projects\WRAIR\WRAIR 2435\Statistics\Programs\Primary Programs\TLF\l_ae.sas 01OCT2019 14:05:50

Listing 16.2.7.1
All Adverse Events
All Enrolled Subjects

Subject ID	Actual Treatment/ Solicited?	Adverse Event/ System Organ Class/ Preferred Term	Onset Date/ Resolution Date or Ongoing	Was Event Serious?	Reason for Seriousness[1]	Severity	Relationship to Study Drug	Action Taken with Study Drug	Outcome[2]
2435-051	CQ/ Yes	PRURITUS/ Skin and subcutaneous tissue disorders/ Pruritus	2018-10-04/ 2018-10-04	No		Mild	Definitely Related	Dose Not Changed	3
	CQ/ No	Sinus Congestion/ Respiratory, thoracic and mediastinal disorders/ Sinus congestion	2018-10-03/ 2018-10-05	No		Moderate	Not Related	Dose Not Changed	3
2435-052	CQ/AZ/ Yes	CHILLS/ General disorders and administration site conditions/ Chills	2018-10-18/ 2018-10-18	No		Mild	Not Related	Dose Not Changed	3
	CQ/AZ/ Yes	DIARRHEA/ Gastrointestinal disorders/ Diarrhoea	2018-09-24/ 2018-09-24	No		Mild	Probably Related	Dose Not Changed	3
	CQ/AZ/ Yes	DIARRHEA/ Gastrointestinal disorders/ Diarrhoea	2018-10-15/ 2018-10-15	No		Mild	Probably Related	Dose Not Changed	3
	CQ/AZ/ Yes	NAUSEA/ Gastrointestinal disorders/ Nausea	2018-10-15/ 2018-10-15	No		Mild	Probably Related	Dose Not Changed	3
	CQ/AZ/ No	Tachycardia/ Cardiac disorders/ Tachycardia	2018-09-24/ 2018-10-01	No		Mild	Not Related	Dose Not Changed	3

Abbreviations: CQ/AZ = Chloroquine-Azithromycin; CQ = Chloroquine.
Note: CQ/AZ group receives 300 mg of Chloroquine and 2 g of Azithromycin orally weekly. CQ group receives 300 mg of Chloroquine orally weekly. Adverse Events are coded using Medical Dictionary for Regulatory Activities Version 21.1.
[1] 1 = Death; 2 = Life-Threatening; 3 = Requires inpatient hospitalization or prolongation of existing hospitalization; 4 = Results in persistent or significant disability/incapacity; 5 = Congenital anomaly/birth defect; 6 = Other medically important event.
[2] 1 = Fatal; 2 = Not Recovered/Not Resolved; 3 = Recovered/Resolved; 4 = Recovered/Resolved with Sequelae; 5 = Recovering/Resolving; 6 = Unknown.
Source: SDC-P:\Projects\WRAIR\WRAIR 2435\Statistics\Programs\Primary Programs\TLF\l_ae.sas 01OCT2019 14:05:50

Listing 16.2.7.1
All Adverse Events
All Enrolled Subjects

Subject ID	Actual Treatment/ Solicited?	Adverse Event/ System Organ Class/ Preferred Term	Onset Date/ Resolution Date or Ongoing	Was Event Serious?	Reason for Seriousness[1]	Severity	Relationship to Study Drug	Action Taken with Study Drug	Outcome[2]
2435-055	CQ/AZ/ Yes	ABDOMINAL PAIN/ Gastrointestinal disorders/ Abdominal pain	2018-10-01/ 2018-10-01	No		Mild	Probably Related	Dose Not Changed	3

Abbreviations: CQ/AZ = Chloroquine-Azithromycin; CQ = Chloroquine.
Note: CQ/AZ group receives 300 mg of Chloroquine and 2 g of Azithromycin orally weekly. CQ group receives 300 mg of Chloroquine orally weekly. Adverse Events are coded using Medical Dictionary for Regulatory Activities Version 21.1.
[1] 1 = Death; 2 = Life-Threatening; 3 = Requires inpatient hospitalization or prolongation of existing hospitalization; 4 = Results in persistent or significant disability/incapacity; 5 = Congenital anomaly/birth defect; 6 = Other medically important event.
[2] 1 = Fatal; 2 = Not Recovered/Not Resolved; 3 = Recovered/Resolved; 4 = Recovered/Resolved with Sequelae; 5 = Recovering/Resolving; 6 = Unknown.
Source: SDC-P:\Projects\WRAIR\WRAIR 2435\Statistics\Programs\Primary Programs\TLF\l_ae.sas 01OCT2019 14:05:50

Listing 16.2.7.1
All Adverse Events
All Enrolled Subjects

Subject ID	Actual Treatment/ Solicited?	Adverse Event/ System Organ Class/ Preferred Term	Onset Date/ Resolution Date or Ongoing	Was Event Serious?	Reason for Serious-ness[1]	Severity	Relationship to Study Drug	Action Taken with Study Drug	Outcome[2]
2435-055	CQ/AZ/ Yes	DIARRHEA/ Gastrointestinal disorders/ Diarrhoea	2018-10-15/ 2018-10-15	No		Mild	Probably Related	Dose Not Changed	3
	CQ/AZ/ No	Fracture to right 2nd m/ Injury, poisoning and procedural complications/ Foot fracture	2018-10-25/ 2018-11-23	No		Mild	Not Related	Not Applicable	3
	CQ/AZ/ No	Muscle Strain/ Injury, poisoning and procedural complications/ Muscle strain	2018-09-28/ 2018-10-10	No		Mild	Not Related	Dose Not Changed	3
2435-056	CQ/AZ/ No	Arm Muscle Pain/ Musculoskeletal and connective tissue disorders/ Myalgia	2018-10-19/ 2018-10-21	No		Mild	Not Related	Dose Not Changed	3
	CQ/AZ/ Yes	HEADACHE/ Nervous system disorders/ Headache	2018-12-13/ 2018-12-13	No		Mild	Unlikely Related	Not Applicable	3
	CQ/AZ/ No	Hives/ Skin and subcutaneous tissue disorders/ Urticaria	2018-10-24/ 2018-10-25	No		Mild	Unlikely Related	Dose Not Changed	3
	CQ/AZ/ No	Hypersensitivity Reacti/ Immune system disorders/ Allergy to arthropod bite	2018-10-04/ 2018-10-09	No		Mild	Not Related	Dose Not Changed	3

Abbreviations: CQ/AZ = Chloroquine-Azithromycin; CQ = Chloroquine.
Note: CQ/AZ group receives 300 mg of Chloroquine and 2 g of Azithromycin orally weekly. CQ group receives 300 mg of Chloroquine orally weekly. Adverse Events are coded using Medical Dictionary for Regulatory Activities Version 21.1.
[1] 1 = Death; 2 = Life-Threatening; 3 = Requires inpatient hospitalization or prolongation of existing hospitalization; 4 = Results in persistent or significant disability/incapacity; 5 = Congenital anomaly/birth defect; 6 = Other medically important event.
[2] 1 = Fatal; 2 = Not Recovered/Not Resolved; 3 = Recovered/Resolved; 4 = Recovered/Resolved with Sequelae; 5 = Recovering/Resolving; 6 = Unknown.
Source: SDC-P:\Projects\WRAIR\WRAIR 2435\Statistics\Programs\Primary Programs\TLF\l_ae.sas 01OCT2019 14:05:50

Listing 16.2.7.1
All Adverse Events
All Enrolled Subjects

Subject ID	Actual Treatment/ Solicited?	Adverse Event/ System Organ Class/ Preferred Term	Onset Date/ Resolution Date or Ongoing	Was Event Serious?	Reason for Serious-ness[1]	Severity	Relationship to Study Drug	Action Taken with Study Drug	Outcome[2]
	CQ/AZ/ Yes	MYALGIA/ Musculoskeletal and connective tissue disorders/ Myalgia	2018-10-15/ 2018-10-15	No		Mild	Not Related	Dose Not Changed	3
	CQ/AZ/ No	Malaria/ Infections and infestations/ Malaria	2018-11-04/ 2018-11-17	No		Severe	Unlikely Related	Not Applicable	3

Abbreviations: CQ/AZ = Chloroquine-Azithromycin; CQ = Chloroquine.
Note: CQ/AZ group receives 300 mg of Chloroquine and 2 g of Azithromycin orally weekly. CQ group receives 300 mg of Chloroquine orally weekly. Adverse Events are coded using Medical Dictionary for Regulatory Activities Version 21.1.
[1] 1 = Death; 2 = Life-Threatening; 3 = Requires inpatient hospitalization or prolongation of existing hospitalization; 4 = Results in persistent or significant disability/incapacity; 5 = Congenital anomaly/birth defect; 6 = Other medically important event.
[2] 1 = Fatal; 2 = Not Recovered/Not Resolved; 3 = Recovered/Resolved; 4 = Recovered/Resolved with Sequelae; 5 = Recovering/Resolving; 6 = Unknown.
Source: SDC-P:\Projects\WRAIR\WRAIR 2435\Statistics\Programs\Primary Programs\TLF\l_ae.sas 01OCT2019 14:05:50

Listing 16.2.7.1
All Adverse Events
All Enrolled Subjects

Subject ID	Actual Treatment/ Solicited?	Adverse Event/ System Organ Class/ Preferred Term	Onset Date/ Resolution Date or Ongoing	Was Event Serious?	Reason for Seriousness[1]	Severity	Relationship to Study Drug	Action Taken with Study Drug	Outcome[2]
2435-056	CQ/AZ/ Yes	NAUSEA/ Gastrointestinal disorders/ Nausea	2018-10-15/ 2018-10-15	No		Mild	Probably Related	Dose Not Changed	3
	CQ/AZ/ Yes	NAUSEA/ Gastrointestinal disorders/ Nausea	2018-12-13/ 2018-12-13	No		Mild	Unlikely Related	Not Applicable	3
	CQ/AZ/ Yes	PRURITUS/ Skin and subcutaneous tissue disorders/ Pruritus	2018-10-04/ 2018-10-04	No		Mild	Unlikely Related	Dose Not Changed	3
	CQ/AZ/ Yes	PRURITUS/ Skin and subcutaneous tissue disorders/ Pruritus	2018-10-24/ 2018-10-25	No		Mild	Unlikely Related	Dose Not Changed	3
	CQ/AZ/ No	Upper Respiratory Infec/ Infections and infestations/ Upper respiratory tract infection	2018-10-31/ 2018-11-04	No		Moderate	Not Related	Not Applicable	3
	CQ/AZ/ Yes	VOMITING/ Gastrointestinal disorders/ Vomiting	2018-10-15/ 2018-10-15	No		Mild	Probably Related	Dose Not Changed	3
2435-057	CQ/ Yes	ABDOMINAL PAIN/ Gastrointestinal disorders/ Abdominal pain	2018-10-12/ 2018-10-12	No		Mild	Not Related	Dose Not Changed	3

Abbreviations: CQ/AZ = Chloroquine-Azithromycin; CQ = Chloroquine.
Note: CQ/AZ group receives 300 mg of Chloroquine and 2 g of Azithromycin orally weekly. CQ group receives 300 mg of Chloroquine orally weekly. Adverse Events are coded using Medical Dictionary for Regulatory Activities Version 21.1.
[1] 1 = Death; 2 = Life-Threatening; 3 = Requires inpatient hospitalization or prolongation of existing hospitalization; 4 = Results in persistent or significant disability/incapacity; 5 = Congenital anomaly/birth defect; 6 = Other medically important event.
[2] 1 = Fatal; 2 = Not Recovered/Not Resolved; 3 = Recovered/Resolved; 4 = Recovered/Resolved with Sequelae; 5 = Recovering/Resolving; 6 = Unknown.
Source: SDC-P:\Projects\WRAIR\WRAIR 2435\Statistics\Programs\Primary Programs\TLF\l_ae.sas 01OCT2019 14:05:50

Listing 16.2.7.1
All Adverse Events
All Enrolled Subjects

Subject ID	Actual Treatment/ Solicited?	Adverse Event/ System Organ Class/ Preferred Term	Onset Date/ Resolution Date or Ongoing	Was Event Serious?	Reason for Seriousness[1]	Severity	Relationship to Study Drug	Action Taken with Study Drug	Outcome[2]
	CQ/ No	Hypersensitivity to Mos/ Immune system disorders/ Allergy to arthropod bite	2018-10-08/ 2018-10-09	No		Mild	Not Related	Dose Not Changed	3
	CQ/ Yes	MYALGIA/ Musculoskeletal and connective tissue disorders/ Myalgia	2018-10-10/ 2018-10-15	No		Moderate	Not Related	Dose Not Changed	3

Abbreviations: CQ/AZ = Chloroquine-Azithromycin; CQ = Chloroquine.
Note: CQ/AZ group receives 300 mg of Chloroquine and 2 g of Azithromycin orally weekly. CQ group receives 300 mg of Chloroquine orally weekly. Adverse Events are coded using Medical Dictionary for Regulatory Activities Version 21.1.
[1] 1 = Death; 2 = Life-Threatening; 3 = Requires inpatient hospitalization or prolongation of existing hospitalization; 4 = Results in persistent or significant disability/incapacity; 5 = Congenital anomaly/birth defect; 6 = Other medically important event.
[2] 1 = Fatal; 2 = Not Recovered/Not Resolved; 3 = Recovered/Resolved; 4 = Recovered/Resolved with Sequelae; 5 = Recovering/Resolving; 6 = Unknown.
Source: SDC-P:\Projects\WRAIR\WRAIR 2435\Statistics\Programs\Primary Programs\TLF\l_ae.sas 01OCT2019 14:05:50

Listing 16.2.7.1
All Adverse Events
All Enrolled Subjects

Subject ID	Actual Treatment/ Solicited?	Adverse Event/ System Organ Class/ Preferred Term	Onset Date/ Resolution Date or Ongoing	Was Event Serious?	Reason for Seriousness[1]	Severity	Relationship to Study Drug	Action Taken with Study Drug	Outcome[2]
2435-057	CQ/ No	Malaria/ Infections and infestations/ Malaria	2018-10-14/ 2018-10-15	No		Moderate	Unlikely Related	Dose Not Changed	3
	CQ/ Yes	PRURITUS/ Skin and subcutaneous tissue disorders/ Pruritus	2018-10-04/ 2018-10-08	No		Mild	Possibly Related	Dose Not Changed	3
	CQ/ No	Viral Upper Respiratory/ Infections and infestations/ Viral upper respiratory tract infection	2018-11-29/ ONGOING	No		Mild	Not Related	Not Applicable	5
2435-059	CQ/AZ/ Yes	DIARRHEA/ Gastrointestinal disorders/ Diarrhoea	2018-10-22/ 2018-10-23	No		Mild	Probably Related	Dose Not Changed	3
	CQ/AZ/ Yes	FATIGUE/ General disorders and administration site conditions/ Fatigue	2018-10-12/ 2018-10-13	No		Mild	Unlikely Related	Dose Not Changed	3
	CQ/AZ/ No	Gastroesophageal Reflux/ Gastrointestinal disorders/ Gastrooesophageal reflux disease	2019-04-08/ ONGOING	No		Mild	Unlikely Related	Not Applicable	5

Abbreviations: CQ/AZ = Chloroquine-Azithromycin; CQ = Chloroquine.
Note: CQ/AZ group receives 300 mg of Chloroquine and 2 g of Azithromycin orally weekly. CQ group receives 300 mg of Chloroquine orally weekly. Adverse Events are coded using Medical Dictionary for Regulatory Activities Version 21.1.
[1] 1 = Death; 2 = Life-Threatening; 3 = Requires inpatient hospitalization or prolongation of existing hospitalization; 4 = Results in persistent or significant disability/incapacity; 5 = Congenital anomaly/birth defect; 6 = Other medically important event.
[2] 1 = Fatal; 2 = Not Recovered/Not Resolved; 3 = Recovered/Resolved; 4 = Recovered/Resolved with Sequelae; 5 = Recovering/Resolving; 6 = Unknown.
Source: SDC-P:\Projects\WRAIR\WRAIR 2435\Statistics\Programs\Primary Programs\TLF\l_ae.sas 01OCT2019 14:05:50

Listing 16.2.7.1
All Adverse Events
All Enrolled Subjects

Subject ID	Actual Treatment/ Solicited?	Adverse Event/ System Organ Class/ Preferred Term	Onset Date/ Resolution Date or Ongoing	Was Event Serious?	Reason for Seriousness[1]	Severity	Relationship to Study Drug	Action Taken with Study Drug	Outcome[2]
	CQ/AZ/ Yes	HEADACHE/ Nervous system disorders/ Headache	2018-10-05/ 2018-10-05	No		Moderate	Probably Related	Dose Not Changed	3
	CQ/AZ/ No	Malaria/ Infections and infestations/ Malaria	2018-11-03/ 2018-11-29	No		Moderate	Not Related	Not Applicable	3
	CQ/AZ/ Yes	PRURITUS/ Skin and subcutaneous tissue disorders/ Pruritus	2018-10-04/ 2018-10-04	No		Mild	Not Related	Dose Not Changed	3

Abbreviations: CQ/AZ = Chloroquine-Azithromycin; CQ = Chloroquine.
Note: CQ/AZ group receives 300 mg of Chloroquine and 2 g of Azithromycin orally weekly. CQ group receives 300 mg of Chloroquine orally weekly. Adverse Events are coded using Medical Dictionary for Regulatory Activities Version 21.1.
[1] 1 = Death; 2 = Life-Threatening; 3 = Requires inpatient hospitalization or prolongation of existing hospitalization; 4 = Results in persistent or significant disability/incapacity; 5 = Congenital anomaly/birth defect; 6 = Other medically important event.
[2] 1 = Fatal; 2 = Not Recovered/Not Resolved; 3 = Recovered/Resolved; 4 = Recovered/Resolved with Sequelae; 5 = Recovering/Resolving; 6 = Unknown.
Source: SDC-P:\Projects\WRAIR\WRAIR 2435\Statistics\Programs\Primary Programs\TLF\l_ae.sas 01OCT2019 14:05:50

Listing 16.2.7.1
All Adverse Events
All Enrolled Subjects

Subject ID	Actual Treatment/ Solicited?	Adverse Event/ System Organ Class/ Preferred Term	Onset Date/ Resolution Date or Ongoing	Was Event Serious?	Reason for Seriousness[1]	Severity	Relationship to Study Drug	Action Taken with Study Drug	Outcome[2]
2435-059	CQ/AZ/ No	Right Knee Pain/ Musculoskeletal and connective tissue disorders/ Arthralgia	2018-10-12/ 2018-10-13	No		Mild	Not Related	Dose Not Changed	3
2435-060	CQ/ No	Constipation/ Gastrointestinal disorders/ Constipation	2018-10-18/ 2018-10-28	No		Moderate	Definitely Related	Dose Not Changed	3
	CQ/ No	Malaria/ Infections and infestations/ Malaria	2018-10-18/ 2018-10-22	No		Moderate	Not Related	Drug Withdrawn	3
	CQ/ Yes	PRURITUS/ Skin and subcutaneous tissue disorders/ Pruritus	2018-10-04/ 2018-10-04	No		Mild	Not Related	Dose Not Changed	3
2435-067	CQ/ No	Hypertension/ Vascular disorders/ Hypertension	2018-09-17/ ONGOING	No		Moderate	Unlikely Related	Dose Not Changed	5
	CQ/ No	Malaria/ Infections and infestations/ Malaria	2018-10-15/ 2018-10-19	No		Moderate	Not Related	Drug Withdrawn	3

Abbreviations: CQ/AZ = Chloroquine-Azithromycin; CQ = Chloroquine.
Note: CQ/AZ group receives 300 mg of Chloroquine and 2 g of Azithromycin orally weekly. CQ group receives 300 mg of Chloroquine orally weekly. Adverse Events are coded using Medical Dictionary for Regulatory Activities Version 21.1.
[1]1 = Death; 2 = Life-Threatening; 3 = Requires inpatient hospitalization or prolongation of existing hospitalization; 4 = Results in persistent or significant disability/incapacity; 5 = Congenital anomaly/birth defect; 6 = Other medically important event.
[2]1 = Fatal; 2 = Not Recovered/Not Resolved; 3 = Recovered/Resolved; 4 = Recovered/Resolved with Sequelae; 5 = Recovering/Resolving; 6 = Unknown.
Source: SDC-P:\Projects\WRAIR\WRAIR 2435\Statistics\Programs\Primary Programs\TLF\l_ae.sas 01OCT2019 14:05:50

Listing 16.2.7.1
All Adverse Events
All Enrolled Subjects

Subject ID	Actual Treatment/ Solicited?	Adverse Event/ System Organ Class/ Preferred Term	Onset Date/ Resolution Date or Ongoing	Was Event Serious?	Reason for Seriousness[1]	Severity	Relationship to Study Drug	Action Taken with Study Drug	Outcome[2]
2435-068	CQ/AZ/ No	Loose Stool/ Gastrointestinal disorders/ Diarrhoea	2018-10-01/ 2018-10-01	No		Mild	Probably Related	Dose Not Changed	3
	CQ/AZ/ Yes	NAUSEA/ Gastrointestinal disorders/ Nausea	2018-09-24/ 2018-09-24	No		Mild	Probably Related	Dose Not Changed	3

Abbreviations: CQ/AZ = Chloroquine-Azithromycin; CQ = Chloroquine.
Note: CQ/AZ group receives 300 mg of Chloroquine and 2 g of Azithromycin orally weekly. CQ group receives 300 mg of Chloroquine orally weekly. Adverse Events are coded using Medical Dictionary for Regulatory Activities Version 21.1.
[1] 1 = Death; 2 = Life-Threatening; 3 = Requires inpatient hospitalization or prolongation of existing hospitalization; 4 = Results in persistent or significant disability/incapacity; 5 = Congenital anomaly/birth defect; 6 = Other medically important event.
[2] 1 = Fatal; 2 = Not Recovered/Not Resolved; 3 = Recovered/Resolved; 4 = Recovered/Resolved with Sequelae; 5 = Recovering/Resolving; 6 = Unknown.
Source: SDC-P:\Projects\WRAIR\WRAIR 2435\Statistics\Programs\Primary Programs\TLF\l_ae.sas 01OCT2019 14:05:50

Listing 16.2.7.1
All Adverse Events
All Enrolled Subjects

Subject ID	Actual Treatment/ Solicited?	Adverse Event/ System Organ Class/ Preferred Term	Onset Date/ Resolution Date or Ongoing	Was Event Serious?	Reason for Seriousness[1]	Severity	Relationship to Study Drug	Action Taken with Study Drug	Outcome[2]
2435-068	CQ/AZ/ Yes	NAUSEA/ Gastrointestinal disorders/ Nausea	2018-10-01/ 2018-10-01	No		Mild	Probably Related	Dose Not Changed	3
2435-070	CQ/ Yes	NAUSEA/ Gastrointestinal disorders/ Nausea	2018-09-18/ 2018-09-18	No		Mild	Probably Related	Dose Not Changed	3
2435-074	CQ/ Yes	ABDOMINAL PAIN/ Gastrointestinal disorders/ Abdominal pain	2018-09-23/ 2018-09-23	No		Mild	Possibly Related	Dose Not Changed	3
	CQ/ Yes	DIARRHEA/ Gastrointestinal disorders/ Diarrhoea	2018-09-21/ 2018-09-21	No		Mild	Possibly Related	Dose Not Changed	3
	CQ/ Yes	FATIGUE/ General disorders and administration site conditions/ Fatigue	2018-09-24/ 2018-09-25	No		Mild	Possibly Related	Dose Not Changed	3
	CQ/ No	Hypersensitivity to mos/ Immune system disorders/ Allergy to arthropod bite	2018-10-04/ 2018-10-08	No		Mild	Not Related	Dose Not Changed	3

Abbreviations: CQ/AZ = Chloroquine-Azithromycin; CQ = Chloroquine.
Note: CQ/AZ group receives 300 mg of Chloroquine and 2 g of Azithromycin orally weekly. CQ group receives 300 mg of Chloroquine orally weekly. Adverse Events are coded using Medical Dictionary for Regulatory Activities Version 21.1.
[1] 1 = Death; 2 = Life-Threatening; 3 = Requires inpatient hospitalization or prolongation of existing hospitalization; 4 = Results in persistent or significant disability/incapacity; 5 = Congenital anomaly/birth defect; 6 = Other medically important event.
[2] 1 = Fatal; 2 = Not Recovered/Not Resolved; 3 = Recovered/Resolved; 4 = Recovered/Resolved with Sequelae; 5 = Recovering/Resolving; 6 = Unknown.
Source: SDC-P:\Projects\WRAIR\WRAIR 2435\Statistics\Programs\Primary Programs\TLF\l_ae.sas 01OCT2019 14:05:50

Listing 16.2.7.1
All Adverse Events
All Enrolled Subjects

Subject ID	Actual Treatment/ Solicited?	Adverse Event/ System Organ Class/ Preferred Term	Onset Date/ Resolution Date or Ongoing	Was Event Serious?	Reason for Seriousness[1]	Severity	Relationship to Study Drug	Action Taken with Study Drug	Outcome[2]
	CQ/ No	Malaria/ Infections and infestations/ Malaria	2018-10-15/ 2018-10-19	No		Severe	Unlikely Related	Not Applicable	3
	CQ/ Yes	VAGINAL YEAST INFECTION/ Infections and infestations/ Vulvovaginal mycotic infection	2018-10-19/ 2018-10-20	No		Mild	Possibly Related	Not Applicable	3

Abbreviations: CQ/AZ = Chloroquine-Azithromycin; CQ = Chloroquine.
Note: CQ/AZ group receives 300 mg of Chloroquine and 2 g of Azithromycin orally weekly. CQ group receives 300 mg of Chloroquine orally weekly. Adverse Events are coded using Medical Dictionary for Regulatory Activities Version 21.1.
[1] 1 = Death; 2 = Life-Threatening; 3 = Requires inpatient hospitalization or prolongation of existing hospitalization; 4 = Results in persistent or significant disability/incapacity; 5 = Congenital anomaly/birth defect; 6 = Other medically important event.
[2] 1 = Fatal; 2 = Not Recovered/Not Resolved; 3 = Recovered/Resolved; 4 = Recovered/Resolved with Sequelae; 5 = Recovering/Resolving; 6 = Unknown.
Source: SDC-P:\Projects\WRAIR\WRAIR 2435\Statistics\Programs\Primary Programs\TLF\l_ae.sas 01OCT2019 14:05:50

Listing 16.2.7.1
All Adverse Events
All Enrolled Subjects

Subject ID	Actual Treatment/ Solicited?	Adverse Event/ System Organ Class/ Preferred Term	Onset Date/ Resolution Date or Ongoing	Was Event Serious?	Reason for Seriousness[1]	Severity	Relationship to Study Drug	Action Taken with Study Drug	Outcome[2]
2435-075	CQ/AZ/ Yes	ABDOMINAL PAIN/ Gastrointestinal disorders/ Abdominal pain	2018-10-12/ 2018-10-13	No		Mild	Unlikely Related	Dose Not Changed	3
	CQ/AZ/ Yes	HEADACHE/ Nervous system disorders/ Headache	2018-11-04/ 2018-11-04	No		Mild	Unlikely Related	Dose Not Changed	3
	CQ/AZ/ No	Malaria/ Infections and infestations/ Malaria	2018-11-01/ 2018-11-04	No		Mild	Unlikely Related	Not Applicable	3
2435-077	CQ/AZ/ Yes	HEADACHE/ Nervous system disorders/ Headache	2018-11-15/ 2018-11-15	No		Mild	Not Related	Not Applicable	3
	CQ/AZ/ Yes	NAUSEA/ Gastrointestinal disorders/ Nausea	2018-10-15/ 2018-10-15	No		Mild	Probably Related	Dose Not Changed	3
	CQ/AZ/ Yes	PRURITUS/ Skin and subcutaneous tissue disorders/ Pruritus	2018-10-04/ 2018-10-04	No		Mild	Definitely Related	Dose Not Changed	3

Abbreviations: CQ/AZ = Chloroquine-Azithromycin; CQ = Chloroquine.
Note: CQ/AZ group receives 300 mg of Chloroquine and 2 g of Azithromycin orally weekly. CQ group receives 300 mg of Chloroquine orally weekly. Adverse Events are coded using Medical Dictionary for Regulatory Activities Version 21.1.
[1] 1 = Death; 2 = Life-Threatening; 3 = Requires inpatient hospitalization or prolongation of existing hospitalization; 4 = Results in persistent or significant disability/incapacity; 5 = Congenital anomaly/birth defect; 6 = Other medically important event.
[2] 1 = Fatal; 2 = Not Recovered/Not Resolved; 3 = Recovered/Resolved; 4 = Recovered/Resolved with Sequelae; 5 = Recovering/Resolving; 6 = Unknown.
Source: SDC-P:\Projects\WRAIR\WRAIR 2435\Statistics\Programs\Primary Programs\TLF\l_ae.sas 01OCT2019 14:05:50

Listing 16.2.7.1
All Adverse Events
All Enrolled Subjects

Subject ID	Actual Treatment/ Solicited?	Adverse Event/ System Organ Class/ Preferred Term	Onset Date/ Resolution Date or Ongoing	Was Event Serious?	Reason for Serious-ness[1]	Severity	Relationship to Study Drug	Action Taken with Study Drug	Outcome[2]
	CQ/AZ/ No	Tachycardia/ Cardiac disorders/ Tachycardia	2018-10-24/ 2018-10-25	No		Mild	Unlikely Related	Not Applicable	3
	CQ/AZ/ No	Temporomandibular Joint/ Musculoskeletal and connective tissue disorders/ Temporomandibular joint syndrome	2019-03-01/ ONGOING	No		Moderate	Not Related	Not Applicable	6

Abbreviations: CQ/AZ = Chloroquine-Azithromycin; CQ = Chloroquine.
Note: CQ/AZ group receives 300 mg of Chloroquine and 2 g of Azithromycin orally weekly. CQ group receives 300 mg of Chloroquine orally weekly. Adverse Events are coded using Medical Dictionary for Regulatory Activities Version 21.1.
[1] 1 = Death; 2 = Life-Threatening; 3 = Requires inpatient hospitalization or prolongation of existing hospitalization; 4 = Results in persistent or significant disability/incapacity; 5 = Congenital anomaly/birth defect; 6 = Other medically important event.
[2] 1 = Fatal; 2 = Not Recovered/Not Resolved; 3 = Recovered/Resolved; 4 = Recovered/Resolved with Sequelae; 5 = Recovering/Resolving; 6 = Unknown.
Source: SDC-P:\Projects\WRAIR\WRAIR 2435\Statistics\Programs\Primary Programs\TLF\l_ae.sas 01OCT2019 14:05:50

Listing 16.2.7.1
All Adverse Events
All Enrolled Subjects

Subject ID	Actual Treatment/ Solicited?	Adverse Event/ System Organ Class/ Preferred Term	Onset Date/ Resolution Date or Ongoing	Was Event Serious?	Reason for Seriousness[1]	Severity	Relationship to Study Drug	Action Taken with Study Drug	Outcome[2]
2435-078	CQ/AZ/ Yes	DIARRHEA/ Gastrointestinal disorders/ Diarrhoea	2018-09-17/ 2018-09-18	No		Mild	Probably Related	Dose Not Changed	3
	CQ/AZ/ No	Malaria/ Infections and infestations/ Malaria	2018-10-13/ 2018-10-16	No		Moderate	Unlikely Related	Drug Withdrawn	3
	CQ/AZ/ Yes	NAUSEA/ Gastrointestinal disorders/ Nausea	2018-09-17/ 2018-09-17	No		Mild	Probably Related	Dose Not Changed	3
	CQ/AZ/ Yes	NAUSEA/ Gastrointestinal disorders/ Nausea	2018-09-24/ 2018-09-27	No		Mild	Probably Related	Dose Not Changed	3
	CQ/AZ/ Yes	PRURITUS/ Skin and subcutaneous tissue disorders/ Pruritus	2018-10-04/ 2018-10-05	No		Mild	Not Related	Dose Not Changed	3
2435-080	CQ/AZ/ No	Loose Stool/ Gastrointestinal disorders/ Diarrhoea	2018-10-01/ 2018-10-01	No		Mild	Definitely Related	Dose Not Changed	3
	CQ/AZ/ Yes	PRURITUS/ Skin and subcutaneous tissue disorders/ Pruritus	2018-10-04/ 2018-10-04	No		Mild	Not Related	Dose Not Changed	3

Abbreviations: CQ/AZ = Chloroquine-Azithromycin; CQ = Chloroquine.
Note: CQ/AZ group receives 300 mg of Chloroquine and 2 g of Azithromycin orally weekly. CQ group receives 300 mg of Chloroquine orally weekly. Adverse Events are coded using Medical Dictionary for Regulatory Activities Version 21.1.
[1] 1 = Death; 2 = Life-Threatening; 3 = Requires inpatient hospitalization or prolongation of existing hospitalization; 4 = Results in persistent or significant disability/incapacity; 5 = Congenital anomaly/birth defect; 6 = Other medically important event.
[2] 1 = Fatal; 2 = Not Recovered/Not Resolved; 3 = Recovered/Resolved; 4 = Recovered/Resolved with Sequelae; 5 = Recovering/Resolving; 6 = Unknown.
Source: SDC-P:\Projects\WRAIR\WRAIR 2435\Statistics\Programs\Primary Programs\TLF\l_ae.sas 01OCT2019 14:05:50

Listing 16.2.7.1
All Adverse Events
All Enrolled Subjects

Subject ID	Actual Treatment/ Solicited?	Adverse Event/ System Organ Class/ Preferred Term	Onset Date/ Resolution Date or Ongoing	Was Event Serious?	Reason for Serious-ness[1]	Severity	Relationship to Study Drug	Action Taken with Study Drug	Outcome[2]
	CQ/AZ/ No	Root Canal for tooth pa/ Gastrointestinal disorders/ Toothache	2018-10-31/ 2018-11-01	No		Mild	Not Related	Not Applicable	3
	CQ/AZ/ Yes	VAGINAL YEAST INFECTION/ Infections and infestations/ Vulvovaginal mycotic infection	2018-10-14/ 2018-10-15	No		Mild	Probably Related	Dose Not Changed	3

Abbreviations: CQ/AZ = Chloroquine-Azithromycin; CQ = Chloroquine.
Note: CQ/AZ group receives 300 mg of Chloroquine and 2 g of Azithromycin orally weekly. CQ group receives 300 mg of Chloroquine orally weekly. Adverse Events are coded using Medical Dictionary for Regulatory Activities Version 21.1.
[1] 1 = Death; 2 = Life-Threatening; 3 = Requires inpatient hospitalization or prolongation of existing hospitalization; 4 = Results in persistent or significant disability/incapacity; 5 = Congenital anomaly/birth defect; 6 = Other medically important event.
[2] 1 = Fatal; 2 = Not Recovered/Not Resolved; 3 = Recovered/Resolved; 4 = Recovered/Resolved with Sequelae; 5 = Recovering/Resolving; 6 = Unknown.
Source: SDC-P:\Projects\WRAIR\WRAIR 2435\Statistics\Programs\Primary Programs\TLF\l_ae.sas 01OCT2019 14:05:50
