# Supplementary material for: An open label study of the safety and efficacy of a single dose of weekly chloroquine and azithromycin administered for malaria prophylaxis in healthy adults challenged with 7G8 chloroquine-resistant Plasmodium falciparum in a controlled human malaria infection model
Source: Malar J. 2020 Sep 16;19:336. doi: 10.1186/s12936-020-03409-z (PMC7493140; doi:10.1186/s12936-020-03409-z)
Supplement: Supplementary file 3 — Additional file 3. PK Data Points: Table of CQ, CQm and AZ exposure levels in the CQAZ cohort. [file 12936_2020_3409_MOESM3_ESM.pdf]

Pharmacokinetic Data Points

2435-41				
		CQ	CQm	AZ
Date	Time (hr)	ng/mL	ng/mL	ng/mL
15-Oct-18	0	11.8	6.8	19.1
15-Oct-18	1	47.3	17.0	1,072.9
15-Oct-18	2	73.1	29.1	2,019.5
15-Oct-18	4	79.9	32.2	553.3
15-Oct-18	6	59.9	27.1	351.8
15-Oct-18	10	47.0	21.2	241.0
16-Oct-18	24	48.9	40.8	141.5
17-Oct-18	48	28.1	13.4	75.2
18-Oct-18	72	25.8	20.0	53.9
19-Oct-18	96	16.0	12.9	33.3

2435-44				
		CQ	CQm	AZ
Date	Time (hr)	ng/mL	ng/mL	ng/mL
15-Oct-18	0	13.7	20.7	25.7
15-Oct-18	1	13.8	22.5	120.9
15-Oct-18	2	29.1	32.7	2,471.5
15-Oct-18	4	100.0	72.4	1,143.2
15-Oct-18	6	190.9	89.5	936.8
15-Oct-18	10	62.3	57.6	419.8
16-Oct-18	24	48.1	66.7	192.8
17-Oct-18	48	27.0	32.6	100.1
18-Oct-18	72	26.0	27.5	78.0
19-Oct-18	96	16.9	25.5	47.0

2435-47				
		CQ	CQm	AZ
Date	Time (hr)	ng/mL	ng/mL	ng/mL
15-Oct-18	0	11.6	9.7	24.6
15-Oct-18	1	16.7	9.7	1,158.5
15-Oct-18	2	60.4	20.8	615.9
15-Oct-18	4	66.0	30.4	477.9
15-Oct-18	6	65.0	38.3	506.8
15-Oct-18	10	54.9	34.1	258.6
16-Oct-18	24	43.2	29.2	177.1

17-Oct-18	48	28.0	17.0	114.7
18-Oct-18	72	23.1	15.3	70.7
19-Oct-18	96	18.2	10.6	53.4
2435-49				
		CQ	CQm	AZ
Date	Time (hr)	ng/mL	ng/mL	ng/mL
15-Oct-18	0	8.6	12.0	16.1
15-Oct-18	1	58.4	24.0	515.9
15-Oct-18	2	135.4	58.7	1,612.8
15-Oct-18	4	59.3	43.0	508.2
15-Oct-18	6	51.7	49.5	343.7
15-Oct-18	10	43.8	39.2	227.2
16-Oct-18	24	39.1	44.2	111.9
17-Oct-18	48	23.7	23.6	59.5
18-Oct-18	72	16.4	14.6	33.9
19-Oct-18	96	12.8	16.2	20.3

2435-52				
		CQ	CQm	AZ
Date	Time (hr)	ng/mL	ng/mL	ng/mL
15-Oct-18	0	9.3	4.1	18.1
15-Oct-18	1	9.8	5.6	687.5
15-Oct-18	2	48.7	19.3	2,898.2
15-Oct-18	4	33.6	17.2	530.9
15-Oct-18	6	41.8	23.6	436.7
15-Oct-18	10	34.9	27.4	223.0
16-Oct-18	24	36.7	27.3	163.6
17-Oct-18	48	20.9	14.3	97.8
18-Oct-18	72	20.2	13.5	68.9
19-Oct-18	96	15.1	10.4	38.1

2435-55				
		CQ	CQm	AZ
Date	Time (hr)	ng/mL	ng/mL	ng/mL
15-Oct-18	0	8.4	7.8	20.0
15-Oct-18	1	19.4	14.5	113.4
15-Oct-18	2	65.7	36.3	2,350.8
15-Oct-18	4	47.1	27.9	754.0
15-Oct-18	6	57.9	52.9	582.2
15-Oct-18	10	55.4	38.7	296.8
16-Oct-18	24	42.3	47.9	151.7

17-Oct-18	48	26.8	20.0	84.1
18-Oct-18	72	18.5	17.9	59.7
19-Oct-18	96	15.6	19.3	36.2

2435-56				
		CQ	CQm	AZ
Date	Time (hr)	ng/mL	ng/mL	ng/mL
15-Oct-18	0	14.1	10.4	13.2
15-Oct-18	1	102.6	39.5	1,490.6
15-Oct-18	2	83.4	41.2	1,066.5
15-Oct-18	4	96.1	44.1	589.0
15-Oct-18	6	81.1	42.5	414.0
15-Oct-18	10	66.2	41.4	221.0
16-Oct-18	24	40.8	24.6	99.0
17-Oct-18	48	23.7	14.3	42.7
18-Oct-18	72	21.9	14.2	29.7
19-Oct-18	96	18.1	14.5	22.2

2435-59				
		CQ	CQm	AZ
Date	Time (hr)	ng/mL	ng/mL	ng/mL
15-Oct-18	0	8.7	5.4	16.0
15-Oct-18	1	13.6	7.0	98.3
15-Oct-18	2	24.2	10.2	936.7
15-Oct-18	4	25.5	13.4	225.6
15-Oct-18	6	27.4	16.9	173.9
15-Oct-18	10	29.3	17.2	131.9
16-Oct-18	24	22.8	18.3	70.3
17-Oct-18	48	16.1	9.8	37.4
18-Oct-18	72	13.6	7.6	28.2
19-Oct-18	96	13.4	7.9	29.3

2435-75				
		CQ	CQm	AZ
Date	Time (hr)	ng/mL	ng/mL	ng/mL
15-Oct-18	0	6.7	6.0	10.3
15-Oct-18	1	8.9	5.6	67.2
15-Oct-18	2	79.1	30.1	2,875.6
15-Oct-18	4	59.9	33.3	393.5
15-Oct-18	6	44.6	29.6	216.2
15-Oct-18	10	42.1	29.2	139.2
16-Oct-18	24	26.3	25.8	77.5

17-Oct-18	48	21.8	15.1	49.3
18-Oct-18	72	14.8	10.7	28.2
19-Oct-18	96	11.0	9.5	18.1

2435-77				
		CQ	CQm	AZ
Date	Time (hr)	ng/mL	ng/mL	ng/mL
15-Oct-18	0	12.9	10.3	45.8
15-Oct-18	1	36.5	18.1	904.2
15-Oct-18	2	100.1	49.6	3,719.4
15-Oct-18	4	94.0	49.6	1,441.6
15-Oct-18	6	93.1	69.2	735.0
15-Oct-18	10	61.4	39.1	373.0
16-Oct-18	24	60.5	63.0	294.3
17-Oct-18	48	49.6	47.1	191.7
18-Oct-18	72	28.6	35.2	124.9
19-Oct-18	96	29.3	35.8	105.1

2435-80				
		CQ	CQm	AZ
Date	Time (hr)	ng/mL	ng/mL	ng/mL
15-Oct-18	0	14.9	14.0	19.2
15-Oct-18	1	96.2	33.0	2,178.4
15-Oct-18	2	138.3	62.4	1,272.4
15-Oct-18	4	107.3	65.6	402.8
15-Oct-18	6	85.7	70.8	321.1
15-Oct-18	10	51.4	36.0	185.5
16-Oct-18	24	60.0	80.2	166.1
17-Oct-18	48	32.4	44.0	70.2
18-Oct-18	72	23.7	27.2	49.2
19-Oct-18	96	20.2	31.4	36.9
